# Spatial attention-based CSR-Unet framework for subdural and epidural hemorrhage segmentation and classification using CT images

**DOI:** 10.1186/s12880-024-01455-6

**Published:** 2024-10-22

**Authors:** Nafees Ahmed S, Prakasam P

**Affiliations:** School of Electronics Engineering, Vellore Institute of Technology, Vellore, India

**Keywords:** Deep Learning, Subdural hemorrhage, Epidural hemorrhage, Intracranial hemorrhage, Segmentation, Classification

## Abstract

**Background:**

Automatic diagnosis and brain hemorrhage segmentation in Computed Tomography (CT) may be helpful in assisting the neurosurgeon in developing treatment plans that improve the patient’s chances of survival. Because medical segmentation of images is important and performing operations manually is challenging, many automated algorithms have been developed for this purpose, primarily focusing on certain image modalities. Whenever a blood vessel bursts, a dangerous medical condition known as intracranial hemorrhage (ICH) occurs. For best results, quick action is required. That being said, identifying subdural (SDH) and epidural haemorrhages (EDH) is a difficult task in this field and calls for a new, more precise detection method.

**Methods:**

This work uses a head CT scan to detect cerebral bleeding and distinguish between two types of dural hemorrhages using deep learning techniques. This paper proposes a rich segmentation approach to segment both SDH and EDH by enhancing segmentation efficiency with a better feature extraction procedure. This method incorporates Spatial attention- based CSR (convolution-SE-residual) Unet, for rich segmentation and precise feature extraction.

**Results:**

According to the study’s findings, the CSR based Spatial network performs better than the other models, exhibiting impressive metrics for all assessed parameters with a mean dice coefficient of 0.970 and mean IoU of 0.718, while EDH and SDH dice scores are 0.983 and 0.969 respectively.

**Conclusions:**

The CSR Spatial network experiment results show that it can perform well regarding dice coefficient. Furthermore, Spatial Unet based on CSR may effectively model the complicated in segmentations and rich feature extraction and improve the representation learning compared to alternative deep learning techniques, of illness and medical treatment, to enhance the meticulousness in predicting the fatality.

## Introduction

 ICH, including subarachnoid (SAH), EDH, SDH are associated with a low probability of functional recovery and a high fatality rate [1]. Even though there is not a significant variation in the outcome of the treatments used for EDH and SDH, there is still debate concerning surgical intervention versus conservative treatments [2]. Even for highly experienced personnel, accurate ICH detection among SDH and EDH is a challenging process. In contrast, determining the bleeding site requires a thorough and time-consuming examination of the ICH diagnosis. Therefore, the success of the next treatment procedure depends on an accurate and timely diagnosis. To solve this problem, some researchers are developing neural networks that can recognize hemorrhages from a patient’s cranial CT scan [3].

Radiologists may be able to diagnose ICH more quickly and accurately by utilizing models based on neural network. Because of its quick acquisition time, head CT scans are often considered as first-line treatment for head traumas, strokes, and other intracranial abnormalities in emergency rooms. Deep learning networks are challenged by the difficult task of differentiating between these two forms of cerebral hemorrhages in head CT scans because the former have distinct shapes while the latter have identical shapes, volumes, and locations [4]. The five subtypes of ICH that are identified by their spots located in the brain are SAH, intraventricular (IVH), EDH, intraparenchymal (IPH), and SDH. Epidural bleeding is brought on by injury to the dura mater and skull. Intraventricular bleeding is the name given to bleeding into the brain’s ventricular system, while IPH is the name given to blood pools within the brain tissues. The emergence of the SAH occurs when blood collects underneath the arachnoid and the pia mater. SDH results from blood moving between the arachnoid and dura mater [5].

**Fig. 1:**
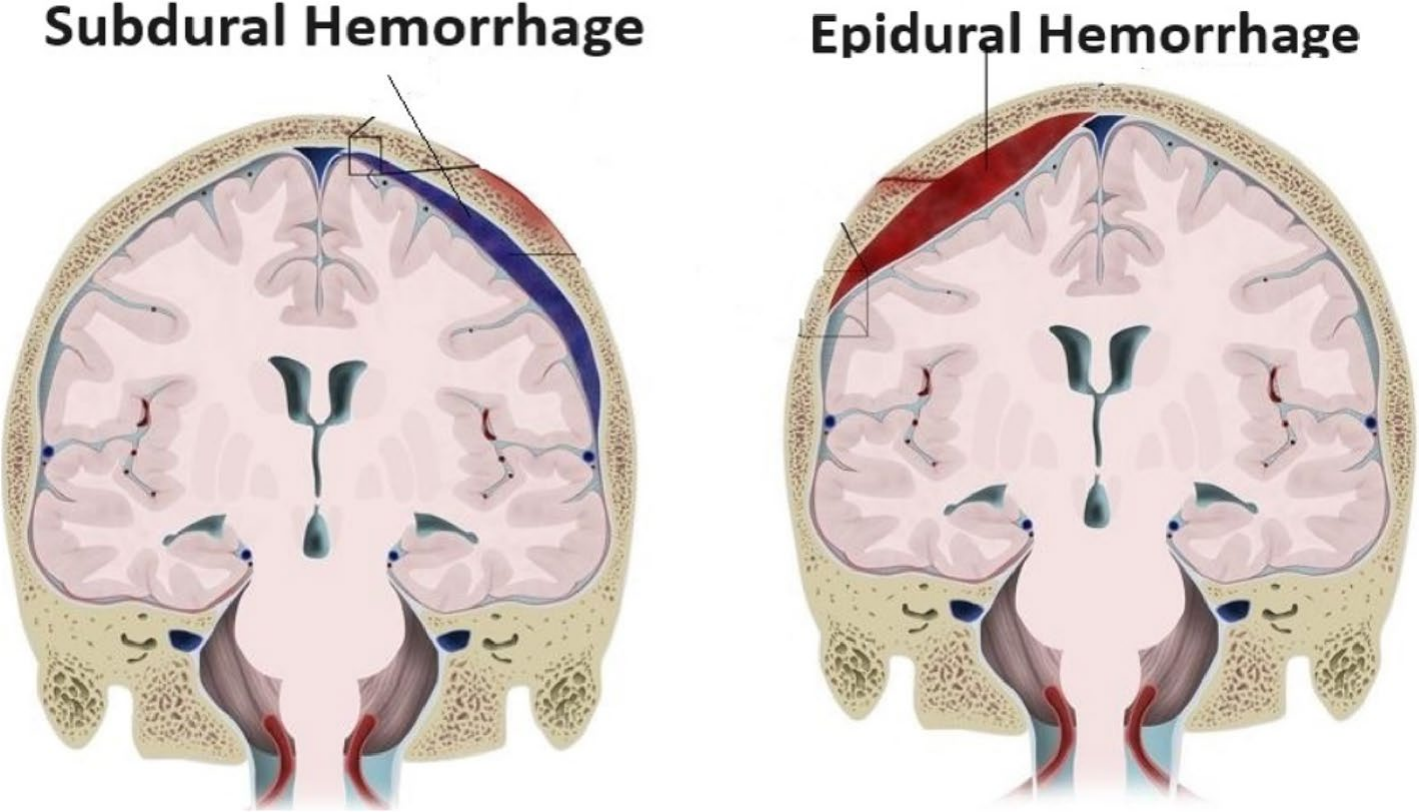
Subdural and Epidural Hemorrhage [5]

Figure 1 illustrates the subdural and epidural types of bleeding, each of which affects different parts of the skull. To enhance the outcomes and lessen the effect of this potentially fatal medical illness, early detection and treatment of ICH are essential [6]. Automatic models are under development to assist doctors in quickly, reliably, and effectively identifying cerebral bleeding lesions from CT scans [7]. Because of their increased processing speed, computer-aided diagnostic (CAD) techniques are now the go-to option for CT image interpretation when compared to human evaluation techniques. The creation of CAD systems revolutionized imaging analysis in the medical field which led to better patient outcomes and more accurate diagnosis. By assisting doctors in making more precise and timely patient diagnoses, these instruments serve as a testament to the advancements made in this field.

The CT scan is often used in critical situations to evaluate patients with ICH [8]. Due to its accessibility and short acquisition time, CT scanning is preferred over magnetic resonance imaging as a diagnostic technique for initial ICH assessment. For medical diagnostics, CT provides the most accurate information on denser tissue with less distortion. Using X-ray beams of differing strengths, CT scans record brain tissues according to the absorption of X-rays, which is expressed in Hounsfield units (HU). The Scan results yield a collection of images.

The type and location of the sickness are determined by a qualified radiologist who assesses these CT scans to determine whether ICH has manifested. The main objective is to differentiate between the area of healthy brain tissue and the area with bleeding brain tissue by breaking up a whole brain CT image into smaller sections. Precise segmentation is necessary for the accurate diagnosis of ICH. The process of segmenting CT scans may be challenging due to the heterogeneous diversity of bleeding patterns, sizes, and locations as well as the existence of noise and anomalies. Early therapy can improve results and lower the risk of side effects like brain damage and disability.

### Motivation

SDHs have been reported in 10–15% of traumatic brain injury cases and can occur in as many as 30% of fatal injuries whereas 5%–15% of cases of severe head injuries are found to be epidural. Despite rapid imaging and transportation, intensive care treatment, and ICP monitoring, A substantial death prevalence of 40–60% and a normal rate of recovery of 19–45% have been correlated with SDH. Mostly affecting the elderly, latest estimates show that 1.5 billion people will be over 65 by 2050, with those over 80 years old comprising the fastest growing cohort increasing percentage of people living in developed nations^1^.

Even though EDH, SDH can be seen in numerous possible space layers outside of the brain, their locations are frequently close to the region of the skull on a CT scan that led to an incorrect diagnosis. It is important to note that, according to the surgical consideration criteria, EDH, SDH, and IPH—three of the five subtypes of ICH are associated. Few studies have divided and categorized the two distinct types of bleeding that are incorrectly diagnosed. Because of the noise, artifacts, asymmetrical limitations, and equivalent intensity of pixel regions in CT images, hemorrhage detection is very hard and consumes more time [9]. Additionally, jobs involving manual evaluation and estimate are very operator-dependent and subject to observer fluctuation. In a large-scale healthcare context, errors and delays could occur in labour-intensive process [10]. Furthermore, several examples of misdiagnosis and clinical impact have been documented during the unavailability of experienced doctors, particularly during odd hours [11].

The variety in SDH and EDH’s position, size and brightness/intensity shown in a head CT scan, however, can make automated diagnosis difficult and the training will get harder and take longer as the network gets more complex. An over simplified deep learning network will miss crucial hemorrhagic characteristics which can lower the accuracy of its identification [12]. Significant works have been completed in the past to use deep learning in order to detect hemorrhages, but cerebrovascular event misdiagnosis rates have been recorded with a value of 8.6%, and even peak rates have been noted in some subgroups [13–15].

Therefore, we focused on diagnosing and assessing hemorrhages of the dural type namely SDH, EDH by splitting and classifying the different hemorrhagic patterns in the CT scans with asymmetrical structures. Additionally, an automated diagnosis system makes it possible for earlier detection, informing radiologists to give the imaging study a higher priority that seeks to cope with the increasing demand for more accurate image segmentation and also addresses the issue of misclassifying SDH as EDH and vice versa.

### Research contributions

The contributions that are vital in the proposed research are as follows.

- Proposed a Spatial attention-based CSR Unet framework that uses a deep learning model which is tailored to accurately segment and classify ICH in CT images.
  - The suggested framework improves the interpretation and assessment of CT scans for the diagnosis of bleeding diseases by incorporating several pre-processing techniques, including resizing, CLAHE, gamma correction, etc.
  - To address the minority class imbalance issues in the dataset, the SMOTE-based class imbalance- solving technique was applied.
  - Proposed Spatial attention-based CSR UNet model for the accurate segmentation and diagnosis of ICH areas in CT images.
- Perform effective extraction of target semantic information with minimal computational demand and to retain the important target details.
- Provide a fully connected layer, convolution layer, and CBL layers as a classifier that uses UNet to categorize dural types of hemorrhages.
- Assess the performance of the suggested Spatial attention-based CSR Unet framework using the metrics: Recall, Precision, F1 score, AUC-RoC and classification accuracy, with Dice Coefficient and IoU for segmentation and compare its performance.

## Related work

Deep learning technology has advanced remarkably in the medical industry in recent years, and it is now a hotspot for drug development, medical image analysis, signal processing research. Since deep learning models are more accurate and versatile, they can handle a wider range of medical images with varying quality and hold an immense potential for drug research where its impact can be found in [14, 15] since they can extract valuable information from challenging molecular systems, fingerprints and carry out tasks that are impossible with conventional computing methods. Furthermore, the deep learning techniques for segmenting various bodily parts and organs in US images are given by Ansari et al. [16].

The authors in [17] conducted a thorough critical analysis of the GAN-based color loss methodology for the creation of realistic EUS images that include both quantitative and qualitative information to help diagnose breast lesions. To achieve real-time liver US segmentation, Dakua et al. [18] suggested the Dense-PSP-UNet, which outperformed the conventional U-Net and other models. Even in signal processing, deep learning plays a vital role in determining age, gender from ECG data and error reduction in signals [19, 20].

Segmentation plays a crucial role in accurately identifying the location of bleeding in each of the brain’s regions and has a stronger medical significance in an ICH image analysis. Medical diagnostic images have been evaluated using a variety of segmentation approaches.

The first artificial neurons were identified in 1943 by McCulloch and Pitts [21]. This technique was further improved upon and applied as technology and procedures developed, becoming a ground-breaking technique for image analysis in various industries. To distinguish ICH from conventional machine learning algorithms, Yuh et al. [22] designed a threshold-based method. Based on the ICH subtypes’ position, volume, shape, the technique was able to identify them. Researchers enhanced the threshold value using retrospective samples of thirty-three CT images. They subsequently tested the model with 210 CT images of patients with brain injuries. With this method, they diagnosed the subtypes of ICH with moderate accuracy, 59% specificity, and 98% sensitivity. A bleeding identification technique was suggested by Shahangian et al. [23] that uses form and texture data in addition to a variation of distance-regularized level set evolution to identify and extract these spots. When there are clear borders, like in the case of EDH hemorrhage types, this approach was considered for the performance, which attained a similarity rate higher than 75%. However, it failed to perform with other hemorrhage categories like SDH, where it only reached a similarity rate of 40%.

Recently, there has been a significant spike in deep learning applications for medical image analysis [24–27]. In [28], deep learning techniques are described for the automatic classification and detection of acute ICH. Using CNN classifiers as an initial framework, this approach uses artificial intelligence (AI) to identify acute ICH. Sequence Models 1, 2, and a recurrent neural network with three-dimensional slices are then used to extract key features for acute ICH detection based on the RSNA, CQ500, and Physionet-ICH datasets—researchers classified ICH in this study. In contrast to other forms of ICH, EDH can be identified with a high degree of sensitivity, considering the results. Furthermore, while utilizing the 2019 RSNA datasets, the suggested approach offers superior classification accuracy compared to the other two datasets. This work has substantial constraints because of the model’s complexity and the length of the training procedure.

Gencturk et al.‘s [29] combination of the Mask Scoring R-CNN and EfficientNet-B2 architecture was used for the identification and categorization of brain hemorrhages. The model provided a two-step verification procedure that improves precision and accuracy. Two different datasets have been used to assess the model’s performance under patient-based and random partitioning strategies, both private and public. The suggested model for SDH hemorrhages in patient-based evaluation has an accuracy of 90% on a private dataset and 91.59% on an open dataset. Real-time CT scans of ICH victims and healthy volunteers were used in a study by Lee et al. [30]. EDH, SAH, and IPH are the three forms of ICH that were categorized with deep learning algorithms that utilize the Kim-Monte Carlo method. 91.7% accuracy in SAH was attained, whereas the overall accuracy rate was 69.6%. This study’s main flaw is that a larger sample size was required to validate the results.

Chang et al.‘s evaluation [31] assessed the accuracy of a customized 3D/2D mask ROI-based CNN in identifying SAH, SDH, EDH, and IPH on non-contrast scans. The AI technique exhibited potential for automated bleeding detection and quantification after being trained on the CT database of a single organization. Deng et al. [32] suggested a model to increase the diagnostic accuracy of EDH and SDH using convolutional neural networks (CNNs). By changing the fundamental model’s parameters and refining with the validation dataset, an ideal model was produced. F1-measure, Precision, and Recall were assessed using the test set. The model’s classification accuracy for the validation set was 0.984, and its average evaluation time for the image was 5.6 s.

Using U-Net and CapsNet on CT images exhibiting the stroke symptoms, Maya et al. [33] divided the hemorrhage region into three types: EDH, IPH, and SDH. Initially, the images included in the study underwent a few different image processing techniques. U-net was then used to segment the data. The segmentation yielded a 75% Dice coefficient. Using CapsNet, segmented images were categorized. CapsNet was able to reach 92.1% validation accuracy. A deep learning method dubbed RADnet based on bi-directional Long Short-Term Memory (LSTM) and DenseNet, was introduced by Grewal et al. [34]. When this method was compared against radiologists (senior), researchers discovered that RADnet is accurate in its predictions similar to radiologists, with enhanced sensitivity, looking for life-saving diagnostic equipment. To direct the model’s attention toward pertinent features and produce segmentation maps that show the extent of bleeding, the team also suggests an additional task: pixel-wise segmentation. The study does, however, warn that further research is required to identify several brain diseases and that RADnet should not be used in place of certified radiologists.

Through automated analysis, Yuh et al. [22] identified whether acute intracranial blood is present in CT scans. Their findings include the existence or non-existence of IPH, SAH, SDH or EDH. Using the thresholding concept, areas with an intensity comparable to blood are first identified. The identified potential region is next classified, taking into account its size, shape, and location, into one of the three categories mentioned above. EDH or SDH are identified when a blood cluster is next to the skull and classified according to the shape and location as IPH or SAH. Nevertheless, this investigation solely considers acute hematoma. ICH can vary in form within the same category, but they can also have comparable, misleading variations in size, shape, and location. Chang et al. [4] provided space and channel attention for more precise extraction of shape and classification of type, as well as an all-attention U-Net to improve class-specific feature extraction to address the issue of SDH and EDH misdiagnosis. The study’s results demonstrated superior performance in situations with restricted attention, with an improvement value of 31.8% was attained when compared with ResNet50 + U-Net.

When it moves to deep learning, the most widely used segmentation models are those that use modified encoder-decoder architecture based on U-Net. UNet [35] achieved remarkable results in the segmentation of medical images via convergent multiscale features, utilizing a symmetric architecture of an encoder and decoder with a skip connection, which was proposed in the year 2015. To confirm the efficaciousness of the deep framework, Xu et al. [36] compared the analysis of the estimation in bleeding volume between the deep model and an approach named ABC/2. They segmented using the Dense U-Net framework. The EDH and SDH dice coefficients for the internal test were 0.88 and 0.82 respectively. Mazahari et al. [37] proposed two convolutional neural networks (CNNs), AlexNet and ResNet50 classifiers, for the detection procedure. The effectiveness of these two architectures was compared, and the optimal model was ultimately suggested and assessed. The segmentation of different kinds of hematoma is becoming more important to experts as intelligent brain hemorrhage segmentation approaches real-world applications. SDH, EDH, and SAH are the multiple forms of cerebral bleeding that Chang et al. [31] proposed to distinguish and isolate utilizing a mask RCN trunk. This technique extracts features using a 3D pyramid network path, and then uses a 2D extended network architecture in order to restore the image quality. Since this method demands high operational resource and several 3D convolution procedures.

An ideal deep learning framework was suggested by Phaphuangwittayakul et al. [38] to support the precise identification of various SDH and EDH subtypes as well as the quantitative evaluation to diagnose ICH. In order to identify the SDH and EDH and extract the important features from the raw input data, they even used a classification neural network which is fine-tuned. In their study, Angkurawaranon et al. [39] analyzed the diagnostic performance of a deep learning model and highlighted the results with identification, localization, and categorization of traumatic ICHs including SDH and EDH. They employed a two-layered, fully connected architecture with four parallel paths processing the input at various resolutions. Regions of SDH and EDH with a main axis lesser than 5 mm were eliminated after segmentation because they appeared noisy. In both cases of SDH and EDH detection, the model’s overall accuracy was 0.89, and corresponding sensitivity, specificity values were 0.82 and 0.90 respectively.

While U-Net + + performs better, its primary drawback is the complexity and amount of effort involved in an execution of significant convolution operations. By maximizing the utilization of feature maps in complete sizes, Humin et al. [40] also introduced the design of U-Net3+, a full-scale connected deep supervision U-Net. SDH, EDH, and SAH are the three forms of cerebral bleeding that Chang et al. [41] proposed to distinguish and separate utilizing a mask RCN trunk. This technique extracts features using a 3D pyramid network path, and then uses a 2D extended network architecture to restore the image resolution. The utilization of several 3D convolution procedures in the method led to the requirement of high operational resources.

### Research Gap

The following drawbacks were identified after an extensive literature study regarding the dural type of hemorrhages (SDH and EDH) in ICH.

- Most of the existing reported methods focused on grouping or classifying subtypes of dural hemorrhage. Only a few researches have been conducted for the two bleeding subtypes regarding categorization and segmentation.
- Traditional machine learning techniques require maximum iterations for convergence, which are expensive to compute and sensitive to initialization.
- Similar texture problems, unclear borders, tiny features, and other constraints hinder the automated segmentation process and reduce its accuracy.
- Radiologists may encounter difficulties in accurately diagnosing dural hemorrhages from CT scans due to the possibility that brain hemorrhage regions could be misinterpreted for calcifications or stripping artifacts.
- Compared to natural images, very small features with tiny border details demand more accurate segmentation in the field of medical imaging.

## Methods

**Fig. 2:**
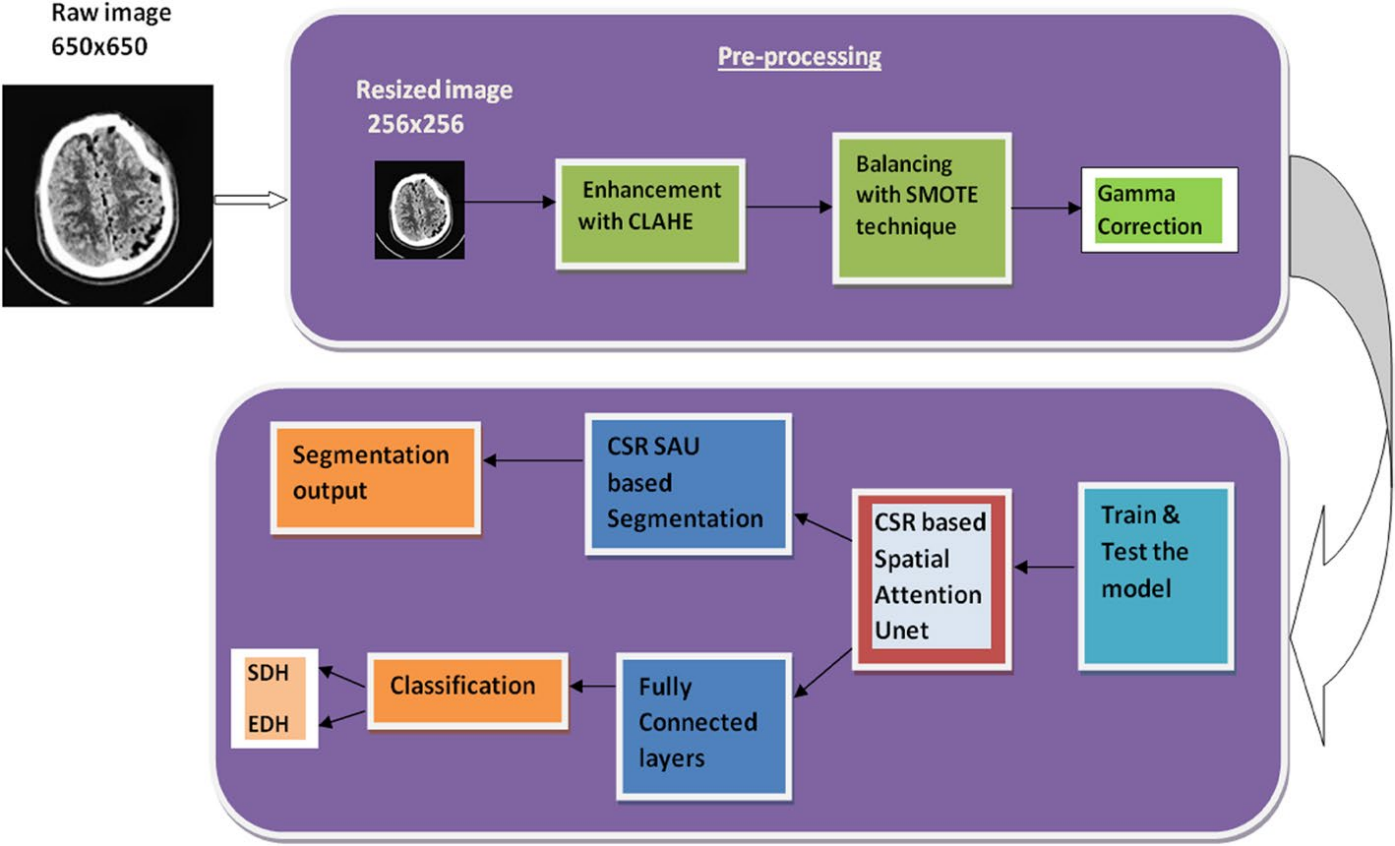
Framework of the proposed model CSR-SAM-UNet

The detailed discussion of the proposed framework for segmentation and classification is illustrated in Fig. 2 and other details of this study are discussed in this given section.

### Dataset

This study made use of a publicly available dataset called Physionet^2^. The public dataset was collected from 82 patients (46 men and 36 women) with traumatic brain injury at Al Hilla Teaching Hospital in Iraq. The average patient age was 27.8 ± 19.5 years, and the CT scans of these patients included 36 patients who had experienced intracranial hemorrhage. In the total of 82 patients, 229 CT scans were related to dural hemorrhages with SDH and EDH counts of 56 and 173 respectively. In addition, no publicly available dataset exists for the ICH segmentation, but numerous publicly available datasets, such as CQ500, RSNA, etc., are available for the ICH classification. ICH segmentation techniques were suggested in other studies in addition to the ICH detection and categorization. Nevertheless, plenty of these methods were not verified because of the absence of their respective ICH masks, hence these disparities and an independent assessment of the various strategies isn’t feasible. Thus, a dataset that can aid in benchmarking and expanding the work is required. The primary goal of this investigation was to collect head CT scans using ICH segmentation along with their respective masks that are accessible in Physionet.

Table 1 highlights the details of Siemens/SOMATOM Definition AS CT scanner specifications.

**Table 1:** CT scanner information

Parameter	Description
Scanner	Siemens
voltage in tubes	100kv
current in tubes	28-500 mA
Range of dynamics	102 db
resolution(isotropic)	0.33 mm
Thickness of slice	5 mm
Interval of slice	5 mm

A CT scan consists of about thirty slices. Of the eighty-two, thirty-six had an ICH of any kind, including IVH, IPH, EDH, SAH, and SDH. Since the number of slices without an ICH was not included in the study, 318 CT slices having an ICH in the dataset were used for training and testing. The dataset shows a notable imbalance in the CT slice count for each subtype of ICH, with most CT scans without an ICH. Furthermore, only five people had an IVH diagnosis, and only four of them experienced an SDH. Every CT slice was initially processed and saved as a 650 by 650 grayscale image.

### Data pre-processing

An image can appear more vibrant and provide more information about the subject of interest by using certain pre-processing techniques. To facilitate a smoother transition for the deep learning network during the training phase, we pre-process the CT scans in our dataset. Since the images in our dataset are not identical to the input images that the deep learning network anticipates to be the same size, the dataset images are resized to a standard size. To lower the amount of computer resources used, each CT slice is reduced from its original dimensions. One other major objective of data preparation is to address data skewness in the training and testing datasets. To do this, each dataset used for training and testing must contain a significant representation of each class in the data that has to be trained. Once every image has been merged into an array and separated into training, validation, and testing datasets, the order of the rearranged dataset is not directly adjustable. This renders the method ineffective. For this reason, it is necessary to divide each class into separate training and testing datasets before combining them [42]. In this proposed research, initially, raw images are resized into 256 × 256 in order to fit perfectly into model’s memory. The resized images are enhanced by using CLAHE technique so that the denoising and improved contrast can help in effective detection. Several denoising techniques have been put forth to lower the image’s noise levels. Nevertheless, these methods result in artifacts and didn’t improve the image’s contrast [43]. Furthermore, CT scans suffer from poor contrast, noise, overlapping boundaries, and differences in the axial rotation [44] which causes difficulty in identifying the hemorrhagic patterns of dural hemorrhages, hence we preferred CLAHE over other denoising techniques. The imbalanced CLAHE- enhanced images were balanced using the SMOTE method which is a superior technique in handling class imbalance issues [15]. SMOTE is preferred in this research as it creates balanced synthetic samples by preserving texture and spatial information which are very crucial, particularly in the dural type of hemorrhages. Finally, the gamma correction is applied to control the intensity of the pixels in the images.

#### Resizing

In order to properly downscale the images as part of the data prior to treatment, it is necessary to analyse the dataset, as a result of which some information can be lost. The ideal image size determined by the tests is recommended in order to preserve memory efficiency and prevent losing any crucial information from the image. Additionally, scaling the image to an extreme large size may surpass the GPU RAM. What standard size is necessary to resize all of our images is the main concern during the resizing process. Either we may choose the largest image size and resize every image to that size, or we can choose the smallest size of an image and resize every image to a size greater than that. During the stretching process, smaller image pixels are forced to stretch by larger image pixels. This may complicate our model’s ability to identify important features like object borders. Stretching is an excellent approach to maximize the number of pixels that are communicated to the network, provided that the input aspect ratio is sufficient.

Proper pre-processing and resizing of the data is essential to attain maximum performance because machine and deep learning algorithms rely heavily on it [45]. It is advantageous to experiment with progressive resizing in order to improve the deep learning network’s training phase, we pre-process the CT images in our dataset. We first examine the trade-off between image size, accuracy, and computing cost, and then we increase the size of the image. In order to get large computational savings and significantly reduce training time, we employed a resizing scale of 256 × 256 pixels. Additionally, the Pillow library function which inbuilt in Python was used to resize the photos while scaling, and no overlap cropping approach was applied. Prior to training the model, the pixels in the image were also normalized from 0 to 1.

#### Contrast Limited Adaptive Histogram Equalization (CLAHE)

Contrast Limited Adaptive Histogram Equalization, or CLAHE for short, is a well-liked image enhancement method, used to enhance and enrich the image’s details. Conventional high enlargement (HE) can increase an image’s overall contrast but tends to weaken small details. The Adaptive Histogram Equalization (AHE) algorithm works better than the HE technique by focusing on certain areas of the image to highlight characteristics. However, there is still room for improvement in the way it handles the transitions between different blocks. The CLAHE algorithm enhances AHE by adding a threshold to control contrast augmentation and minimizes the picture noise. It also performs comprehensive and effective image processing by employing an interpolation technique (linear) to create seamless transitions between blocks of image as the CLAHE algorithm deftly boosts contrast in images [46].

**Fig. 3:**
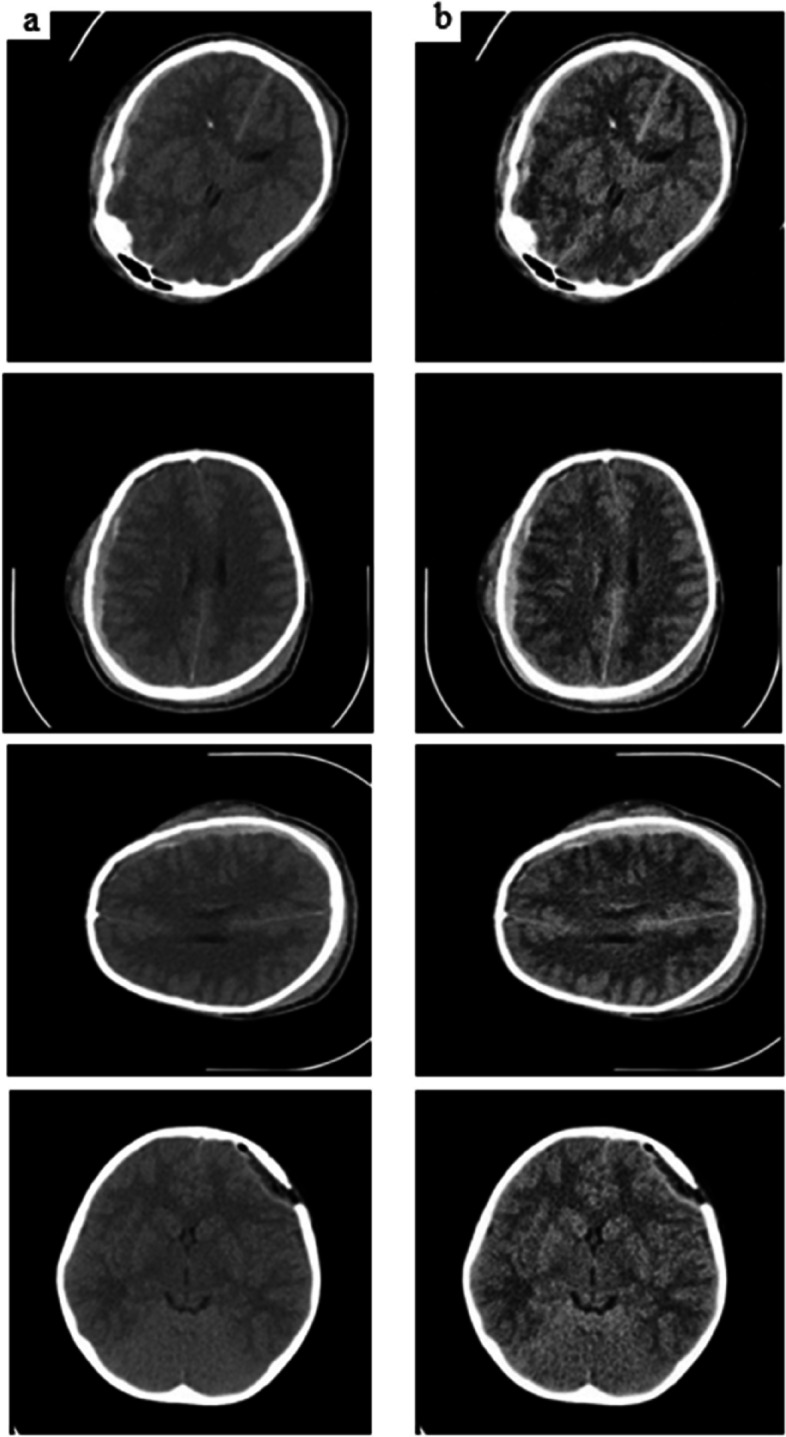
Comparison with enhancement (CLAHE) (**a**) Images before using CLAHE (**b**)Images after using CLAHE

CLAHE also reduces the contrast intensities in areas that can fluctuate, as shown by peaks in the histogram associated with transient zones (i.e., many pixels that fall within the same grayscale), which could potentially reduce noise problems connected to AHE. Figure 3 shows the images both before using and after using of CLAHE enhancement. Slopes related to the gray level assignment method adapted by CLAHE are limited to certain pixel values that are alternatively connected to local histograms. By pixel-by-pixel cropping and retaining count equality, the histogram can be measured fairly. As a result, CLAHE improves image quality and increases efficiency for image processing tasks like object detection, segmentation, and analysis. Image enhancement results in a sharper image and a more precise computational analysis. CLAHE provides an enhanced image deblurring, contrast improvement, and noise reduction [47].

#### Class balancing using SMOTE

The topic of imbalanced classification challenges has attracted a lot of interest. Since it depends on several factors, including the degree of class imbalance, data complexity, dataset size, and the classification technique employed, the performance of the models built from imbalanced datasets is difficult to predict. An imbalanced data is a situation of having an uneven distribution of classes; this is distinct from previous conventional classification problems. This shows that a particular class, often known as the majority class contains more instances than the other class, then the remaining data are termed as the minority class.

**Fig. 4:**
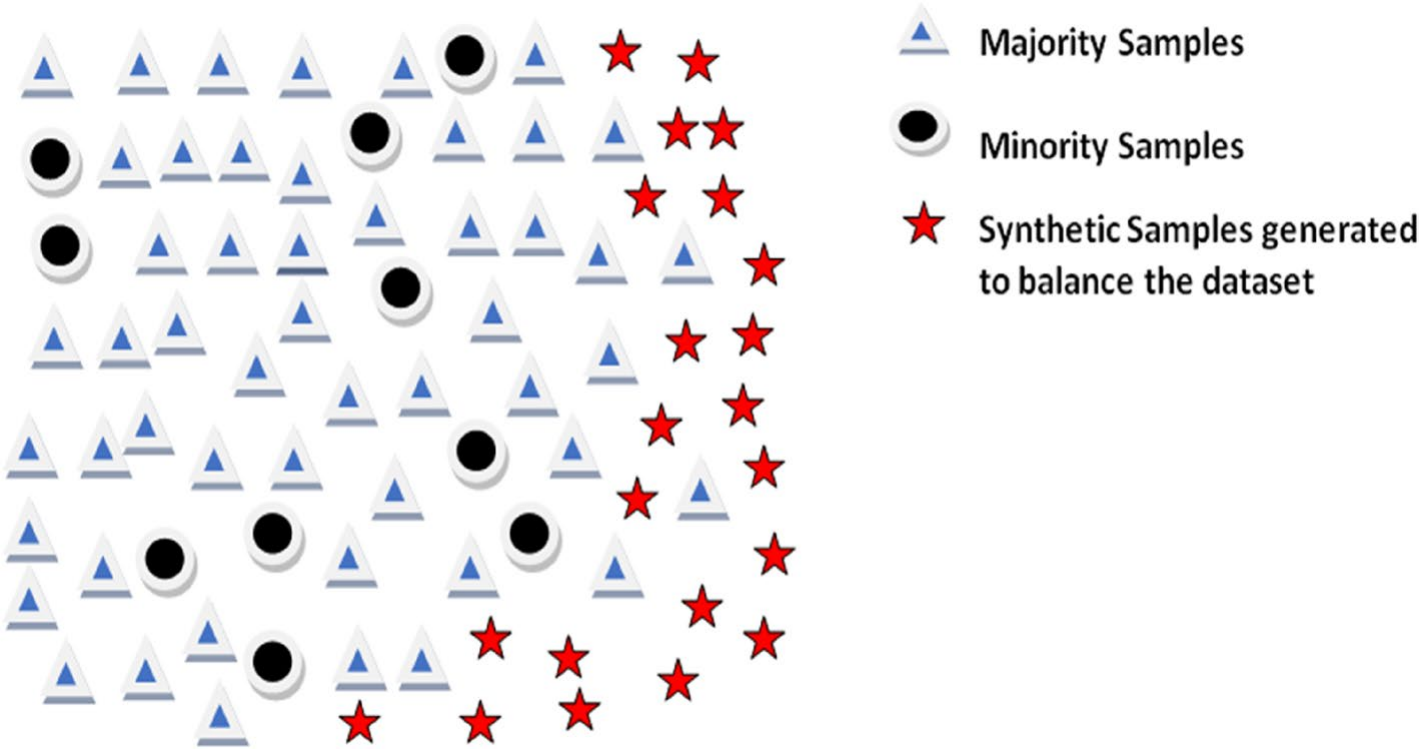
Balancing based on SMOTE

Nevertheless, in these imbalanced tasks, minority class forecasts frequently underperform majority class forecasts, leading to a substantial fraction of minority class predictions that are computed incorrectly. Different approaches to dataset balance are used when these disparities arise. The synthetic minority oversampling technique (SMOTE) was utilized in this study to assess the differences between balanced and unbalanced datasets. Due to their potential to provide better performance with balanced data, SMOTE algorithms are highly effective ways to enhance a model’s capacity for generalization [48]. Unbalanced data can be balanced by using the SMOTE pre-processing approach as shown in Fig. 4.

SMOTE is a non-destructive method that uses linear interpolation to create virtual data points between the existing points of the minority class in order to balance the number of samples in each class. It should be pointed out that while using SMOTE in the case of oversampling, the sensitivity, and specificity are traded off. A better distribution of the training set indicates a surge in the items properly identified for the minority class. It’s an oversampling technique, but it creates new samples through synthesis instead of replicating old ones. In addition to producing samples from underrepresented classes, it offers a balanced dataset. Samples are generated from the line that joins the randomly chosen minority class instance and its nearest neighbors in the SMOTE process.

**Fig. 5:**
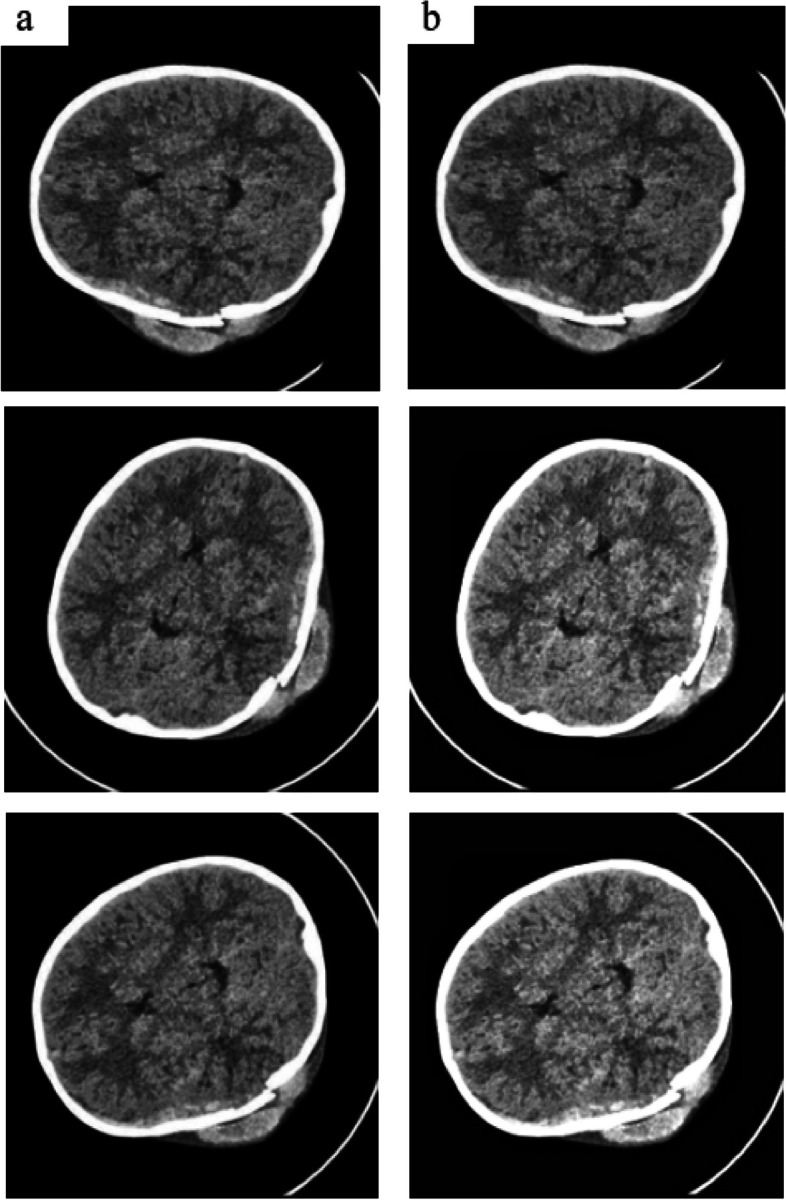
Comparison with SMOTE (**a**) Images before using SMOTE (**b**)Images after using SMOTE

SMOTE is specifically applied to image data by first identifying minority class instances through class label analysis, then choosing nearest neighbours based on distance metrics like Euclidean or cosine similarity, and finally performing interpolation between them to create synthetic samples while maintaining texture, visual characteristics, and spatial information. Furthermore, we applied pre-processing techniques on training images and tested without pre-processing techniques. The sample images before and after applying the SMOTE technique are given in Fig. 5.

We employed a total of 874 images in our investigation, of which 828 were used for training and 46 were used for testing. The total images in Table 2 depicts the number of images before and after using the SMOTE procedure.

**Table 2:** SDH and EDH images before & after using SMOTE technique

Type of ICH	Total number of images	Samples for training before SMOTE (80%)	Samples for testing (20%)	Total training images after applying SMOTE
**SDH**	56	45	11	414
**EDH**	173	138	35	414

#### Gamma correction

The output image’s gray values and the input image’s gray values have an exponential relationship as a result of the nonlinear operation known as gamma correction. In other words, the overall intensity of the image is modified via gamma correction. By changing the power function represented by Ω, gamma correction modifies the intensity of the image as a whole. The features in the highlights are highlighted when Ω < 1, while the details in the shadows are highlighted when Ω > 1. For this reason, gamma corrections and modifying the surface of the object’s reflected light wave drawn the attention of researchers seeking to improve low-light images [49].

### Proposed spatial attention-based CSR-Unet architecture

**Fig. 6:**
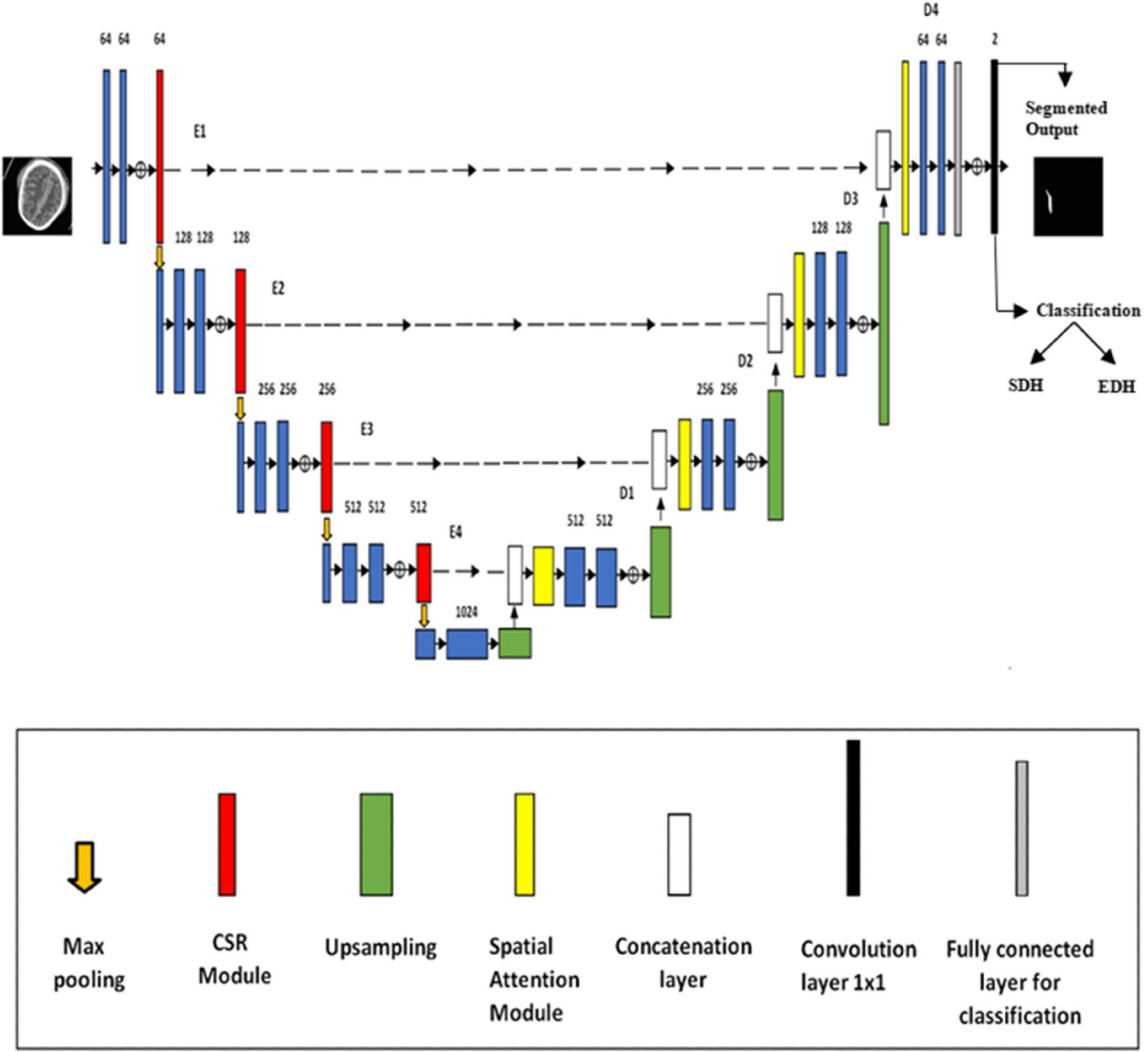
Spatial attention- based CSR-Unet architecture

The general framework of the proposed Spatial Attention based Convolution squeeze excitation residual module (CSR) Unet design, which is based on a U-shaped encoder-decoder network, is depicted in Fig. 6. Our network has improved decoders and encoders, unlike the original U-Net architecture. In order to improve the receptive field and improve segmentation performance, we first decide to optimize each encoder block, sub-sampling block with CSR. E1, E2, E3, and E4 are connected with separate CSR modules that extract the image’s features and aid in extracting information from the input images of the CT slices. The two 3 × 3 convolutional layers that make up E2, E3, and E4 have stride 1 and filters of 128, 256, and 512, respectively. 2 × 2 Max-Pooling, batch normalization, and ReLU activation functions come after each convolutional layer. The following encoder step receives each output from Max-Pooling.

In each down-sampling step, the number of feature channels is doubled and then halved using upsampling. Moreover, CSR blocks are utilized to adaptively extract the image features from the feature map of the encoder convolution in order to obtain rich and precise information [50]. The Squeeze excitation (SE) block’s specific operation before to placing the previously obtained normalized weights to use on each channel’s features, a fully connected neural network and a transformation which is nonlinear are added to the 2D feature map (H x W) of each channel to compress it into necessary features. This process serves the purpose of extracting specific information from each channel and concatenates using the encoder’s associated feature maps.

To ascertain the spatial information, the decoder comprises the D1, D2, D3, and D4 stages. Each decoder step is linked to a spatial attention module which helps in minimizing the resolution loss due to multiple downsampling. This module lowers the parameters while capturing the contextual data of feature maps that have been derived from the encoder stages. Additionally, each stage of the encoder’s extracted features map is sent to the associated decoder by an independently connected CSR module, which incorporates a spatial attention module at each level of the decoder. The output of the Spatial attention module is then fed to the decoder by generating effective feature descriptors, thus the Spatial Attention based CSR Unet when added to the structure to enhance small hemorrhagic feature segmentation through rich feature extraction, feature enhancement, and feature suppression, ultimately improving the network’s representation and improves the segmentation accuracy of small structures.

#### CSR module

**Fig. 7:**
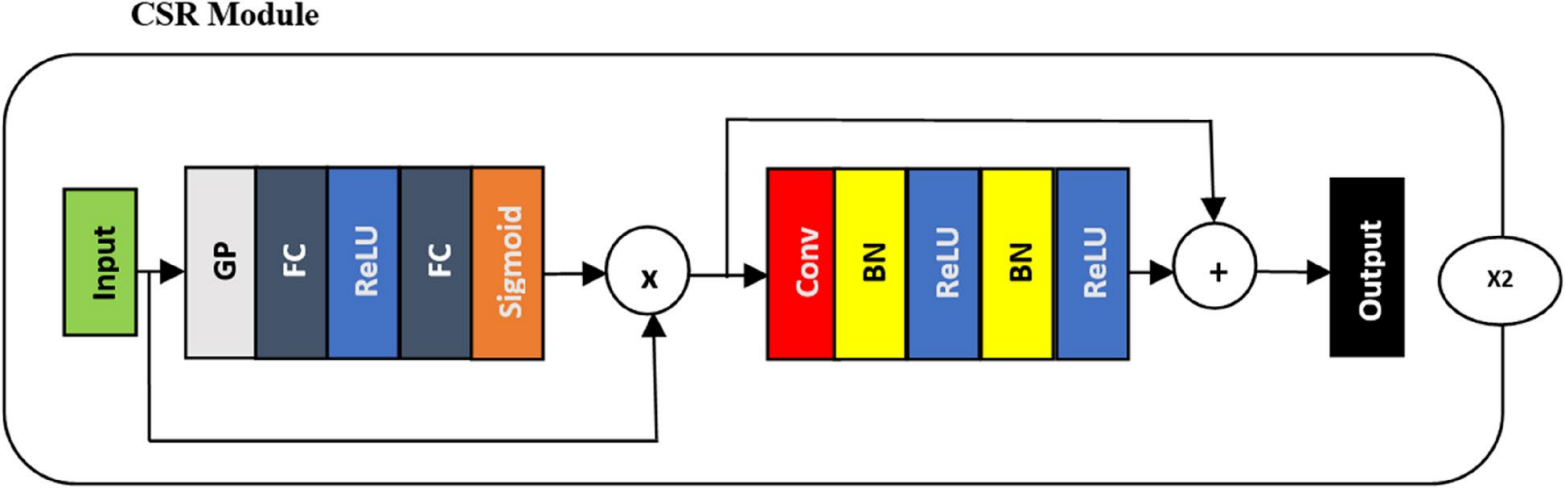
CSR module

CSR module illustrated in Fig. 7 stands for Convolution-Squeeze Excitation Residual involves a residual module for accurate segmentation, a convolution block, and SE (squeeze and excitation) module. One way to think of SE blocks is as feature map channel recalibration modules [51]. The SE module can enhance a model’s long-range dependency modeling capabilities, performance, and ability to generalize in deep learning tasks of image segmentation. During the image segmentation challenge, the SE module helps the model discover the feature map’s various channels’ relevance weights adaptively, improving its ability to represent different image targets. As a result, less focus is given to unimportant data and more attention is directed towards the characteristics that are significant for a certain objective.

By strengthening the model’s capacity for discrimination and generalization, this attention mechanism boosts the model’s performance in tasks of image segmentation. The SE module consists of two main stages, that are called SE. Following a residual convolution block, the input features are first placed through a process called global average pooling, which combines feature information from all of the channels to create a global feature that encodes the spatial characteristics on all of the channels. Subsequently, each channel’s relevance is estimated using a fully connected layer. The resulting feature map is again processed through a rectified linear unit (ReLU) activation function, followed by global average pooling, and sigmoid activation function.

By multiplying the channel weights, the SE module finally completes a sigmoid activation function and global average pooling using a scale operation. Once more, a sigmoid activation function and global average pooling are applied to the resulting feature map following a ReLU activation function. The weight values of each channel calculated by the SE module are ultimately multiplied by each two-dimensional matrix of the corresponding channels of the original feature map by using multiplication with channel weight in order to get the output feature map.

##### Skip connections

There are two types of skip connections that exists in the Fig. 7 namely Skip connection with direct additive and Squeezed type via FC layers.

##### Skip connection with direct additive

This connection runs through from the Conv-BN-ReLU block’s input to its output, avoiding both the sigmoid activation and the block of fully connected (FC) layers. It applies a residual connection, which adds the input straight to the convolutional block’s output. In this instance, the network learns the difference (residual) between the input and the output of the convolutional layers, which facilitates the learning of identity mappings and minor input alterations.

##### Squeezed type via FC layers

Global Pooling (GP), fully connected (FC) layers with ReLU activations, and a final sigmoid activation are the steps in this connection that the input must go through. An attention mechanism (scales the feature maps) adjusts the features learned in the convolutional block as a result of this path. With this connection, the network can learn weights to highlight or suppress specific feature map channels depending on the input. After the fully connected (FC) layers and sigmoid activation in the second skip connection, the channel weights are multiplied by the original feature map. These weights are multiplied element-wise with the feature maps from the Conv-BN-ReLU block. Residual connections typically involve elementwise addition. It enhances the capacity to train deeper networks by preserving dimensionality and feature map structures. The model better understand which feature maps and classes are most relevant to the task by utilizing channel-wise attention. In order to preserve or enhance the most pertinent aspects for future spatial attention tasks, it is necessary to complete this initial phase of channel weighting before the spatial operations [52].

##### Residual network

The first proposal for residual networks was proposed in [53]. Deep learning models perform better on a variety of tasks when there is an adequate depth of network. In theory, the deeper the network, the more accurate the model’s performance should be. Deep networks of this kind, however, could hamper training and could induce a decrease in performance that’s not caused on by overfitting [54]. He and colleagues created residual neural networks that are simple to train in order to address these problems. Several techniques can be used to implement residual units, such as varying combinations of rectified linear unit (ReLU) activation, convolutional layers, and batch normalization (BN). It is necessary to check how various combinations—particularly pre-activation that can cause categorization error, produced by the activation function’s location in relation to the element-wise addition, resulting in post-activation. BN and ReLU are situated before the complete pre-activation. Convolutional layers work well and only affect the residual path in an asymmetric manner. The whole pre-activation residual unit is typically utilized to construct a Residual UNet. Multiple full pre-activation residual units layered in order make up a residual neural network each has the general form of an equation shown below [55].

1 $$\:{\:\:\:\:\:\:\:\:\:\:\:\:y}_{m+1}\:=i\left({y}_{m}\right)+\text{G}(\text{j}\left({y}_{m}\right),{Y}_{m})$$

Where $\:{y}_{m}\:\text{a}\text{n}\text{d}\:{y}_{m+1}$ refers to input, output features of $\:m$ th residual unit, $\:{Y}_{m}$describe the set of biases and weights connected to $\:m.L$ is the number of layers that each residual unit contains$\:{y}_{m}$is a shortcut for a convolution layer that measures 1 × 1 and a BN layer that increases the dimension of $\:{y}_{m}$. The residual function is indicated by $\:\text{G}(\text{j}\left({y}_{m}\right),{Y}_{m})$and $\:\text{i}\left({y}_{m}\right)$ is the ReLU activation function utilized following the BN layer on $\:{y}_{m}$.

##### Spatial attention

Neural network models with attention mechanisms can selectively focus on different segments of input images or sequences. The concept is expanded to focus on relevant spatial areas in an image via spatial attention, a particular kind of attention used in computer vision [56]. By using the spatial associations between features, the spatial attention module—the informative portion aims to create a spatial attention map [57].

**Fig. 8:**
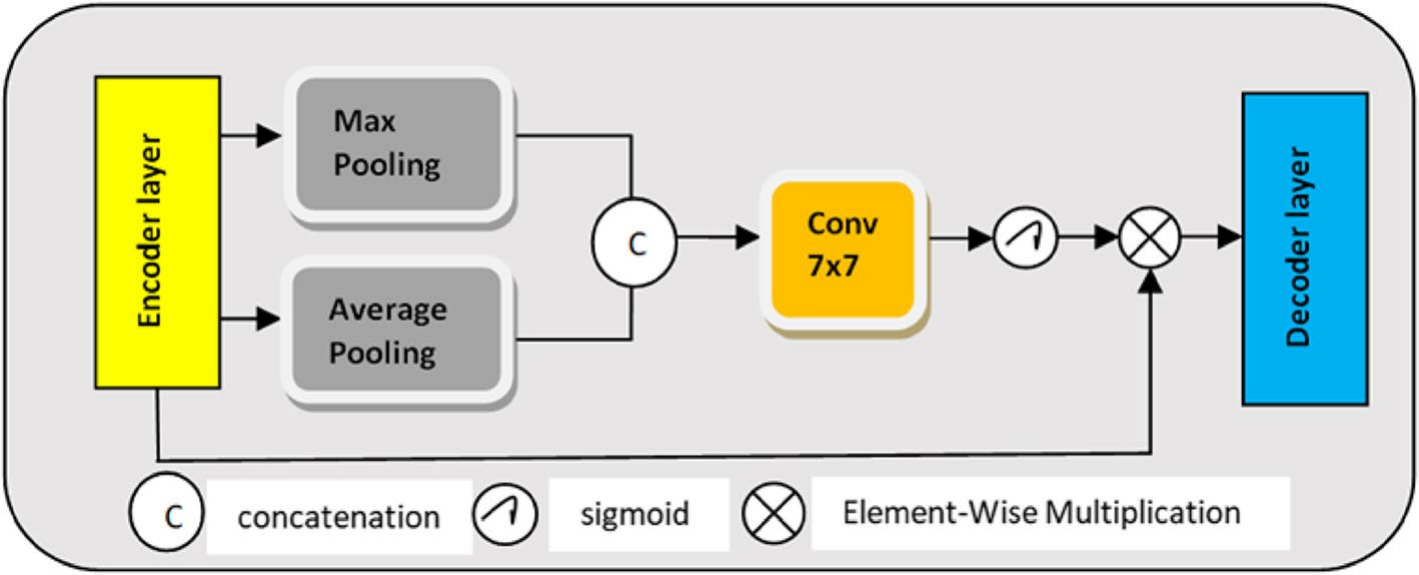
Spatial attention module

The input feature of Spatial attention module is$\:\:\:\text{G}\in\:{\:\text{T}}^{\text{H}\times\:\text{W}\times\:1}$ which is displayed in the Fig. 8 is forwarded via max-pooling (channel-wise) and average pooling for generation of outputs $\:{\text{G}}_{\text{m}\text{a}\text{x}}^{\text{s}}\in\:{\text{T}}^{\text{H}\times\:\text{W}\times\:1}$and $\:{\:\text{G}}_{\text{A}\text{v}\text{g}}^{\text{s}}\in\:{\text{T}}^{\text{H}\times\:\text{W}\times\:1}$. These output feature maps are combined to produce feature descriptors. The convolutional layer with a 7 × 7 kernel size then the sigmoid activation function comes next. Next, a spatial attention map is created by multiplying the output of the sigmoid function layer element-by-element using encoders denoted as $\:{Y}_{SAM}\in\:{T}^{H\times\:W\times\:1}$.

2 $$\:{Y}_{SAM}=\text{G}.{\updelta\:}\left({f}^{7\times\:7}\right(\left[{G}_{max}^{s}\times\:{G}_{Avg}^{s}\right])$$

where$\:{\:f}^{7\times\:7}$ indicates convolution operation with 7 as the kernel size and $\:{\updelta\:}$ denoted Sigmoid function

#### Leaky RELU

There is another drawback identified in the two traditional activation functions, highlighted by deep learning in general and the emergence of deeper architectures. When the network was deep, backpropagation’s limited output restricted the derivatives’ dissipation. In other words, this suggests that the weights of the deeper layers remained relatively constant as they acquired new information throughout training. The cause of this phenomenon is the vanishing gradient problem. To partially solve the difficulties involved in deep learning and computational calculation, the rectified linear unit (ReLU) was developed.

3 $$\:p=max\left\{\:0,q\right\}=q\:|\:q>0$$

ReLU performs exceptionally well while being computationally efficient. Because back-propagation does not restrict positive inputs, it can allow for deeper layer learning, which increases the likelihood that gradients will reach deeper layers. Additionally, the computation of the gradient is reduced to a constant multiplication by backpropagation learning, which results in a much more computationally efficient solution.

Due to its inability to react to negative inputs, the ReLU has a major disadvantage in that it deactivates a large number of neurons during training. Consider this a vanishing gradient problem with negative values. The Leaky rectified linear unit (Leaky ReLU), which partially activates for negative values, is introduced to address the non-activation for non-positive integers.

4 $$\:p\:=\{lq\:\:if\:\:p<0\:\:\:\:\:\:\:\:q\:\:if\:\:q\ge\:0$$

Due to this, the learnable parameter influences both positive and negative values, which is the main advantage of the leaky ReLU. Specifically, it is used to solve the Dying ReLU problem. Think about the idea that l stands for the leak factor. Usually, it is fixed to something incredibly low value like 0.001.

#### Activation function

Softmax can be used in binary classification as well, however it is typically applied to multiclass classification problems. The last activation function that was used was the softmax function. The softmax function takes the output logits, normalizes them into a probability distribution, and converts them into probabilities so that the sum of the output probability is equal to 1. The primary objective is to normalize the output of a network to the probability distribution across the anticipated output classes. This is the traditional Softmax function, denoted by$\:\:X$.

5 $$\:\:\:\:\:\:\:X\left({\text{w}}_{i}\right)=\frac{{e}^{{\text{y}}_{i}}}{{\sum\:}_{m=1}^{l}{e}^{{\text{y}}_{m}}}\:\:for\:\:i=1,\dots\:k\:\:and\:\:\:y=\left({y}_{1},\dots\:{y}_{k}\right)\in\:{S}^{\text{l}}$$

It divides the values by the sum of all these exponentials to normalize them after applying the usual exponential function to each a component $\:{\text{y}}_{\text{i}}$ of the input vector $\:\text{y}$.

#### Loss function

Loss functions are a fundamental part of all deep learning models because they minimize a chosen loss function, which the model uses to determine its weight parameters, and because they provide a standard by which the Spatial Attention-based CSR Unet model’s performance is measured. This experiment’s primary objective is to assess how well loss functions with Dice and focused loss performs. The cross-entropy is utilized to compare the actual class desired output with the expected class probability. A loss is computed to penalize the probability, incurred by variance of the probability from the real expected value.

When the differences are significant and close to 1, the logarithmic penalty produces a large score, when the differences are little and move toward 0, the score becomes small. In this instance, a ground truth class v and a ground truth segmentation target mask w are assigned with labels to each training image input. For each input image, we utilized a multi-task loss L to train the system for both mask segmentation and classification.

6 $$\:\:\:\:\:\:\:\:\:\:\:\:\:\:\:\:\:M={M}_{gl}+{\updelta\:}{M}_{nt}$$

Where $\:{M}_{gl}+{\updelta\:}$ is the balance coefficient and the true class $\:\text{r}$ is the focal loss represented. The output of the segmentation mask branches defines the second task loss, $\:{\text{M}}_{\text{n}\text{t}}$. Typically, we utilize the Dice coefficient—a similarity statistic connected to the Jaccard index—to assess the caliber of image segmentation. The output of the four segmentation mask branches specifies the second task loss, $\:{\text{M}}_{\text{n}\text{t}}$.

The quality of picture segmentation is usually evaluated using the Dice coefficient, a similarity metric related to the Jaccard index. With the loss of dice coefficient being $\:1-{\text{D}}_{\text{j}}$ the coefficient is defined as follows for segmentation output $\:{c}^{{\prime\:}}$and target $\:c$.

7 $$\:{\:\:\:\:\:\:\:\:\:\:\:\:\:\:\:\:\:\:\:\:\:\:\:\:\:\:\:\:\:\:\:\:D}_{j}\left({c}^{{\prime\:}},c\right)=\frac{2\left|c\cap\:{c}^{{\prime\:}}\right|}{\left|c\right|+\left|{c}^{{\prime\:}}\right|}$$

#### Fully connected layers

Fully linked layers in neural networks are the most flexible and are found in almost all design types. Every node is interconnected with other node in the layer above and below it in a totally connected layer. Changing the feature space to make the problem simpler is the main objective of a fully linked layer. Throughout this transition process, the number of dimensions may increase, decrease, or stay the same. Each instance’s new dimensions are linear mixtures of those from the layer above. Next, an activation function is used to introduce non-linearity into the additional dimensions.

Owing to FC layers, any kind of interaction between the input variables is possible. This type of learning without regard to structure allows completely connected layers to theoretically learn any function, given sufficient depth and width. To address this issue, researchers have developed more specialized layers like recurrent and convolutional layers. These layers, which apply inductive bias depending on the spatial or sequential structures of specific data types such as text, photos, etc., basically work like this. In this work, two classes of brain hemorrhages are classified by using the completely connected layers.

The integration of the spatial attention mechanisms, Squeeze-and-Excitation (SE) blocks, and residual connections can significantly increase the computational complexity and memory requirements, since the combination of different modules often requires more time. Furthermore, to tackle this complexity, recent developments have yielded several innovative designs, including multicore, general purpose graphics progressing units (GPGPUs), and field programmable gate arrays, which show tremendous promise for speeding up computationally demanding workloads [58]. Parallelization has been widely utilized since massively computationally intensive procedures are carried out in simulation. However, this additionally suggests that more memory and processing capacity are needed in order to parallelize its iterations [59, 60]. Hence by considering all these challenges and demands of intense computing we trained our models with NVIDIA GeForce RTX 3070 Ti paired to handle the complex deep learning models.

### Proposed model spatial attention based CSR Unet training process

The algorithm demonstrates how the Spatial Attention-based CSR Unet training phase is executed, with parameters such as α1 for the training set, and α2 for the testing set. Meanwhile, the CNN input layer goes through an iteration phase, denoted by δ, which aids in linking the encoder layers E1, E2, E3, and E4 with the CSR layers. Table 3 was used to initialize the parameters of the proposed model. Batch size was assigned, loss functions were calculated, and method 1 illustrated the full process.

**Figure:**
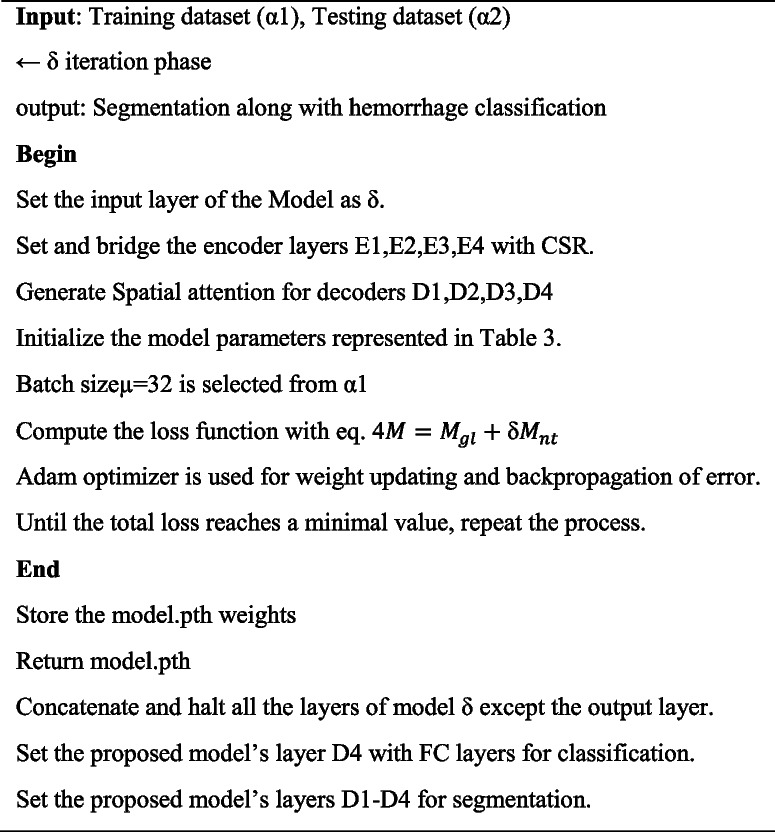
Algorithm 1. Spatial Attention- based CSR Unet training process

**Table 3:** Hyperparameters and preferences

Hyperparameters	Preferences
Image input size	256 × 256
Rate of learning	0.0001
Decline in the Learning Rate Index	0.5
Epochs	60
Batch Size	16
Optimizer	Adam
Loss	Dice
GPU	NVIDIA GeForce RTX 3070Ti

## Performance metrics

Metrics including Accuracy, Specificity, Sensitivity, F1 score, Precision, Recall, IoU and Dice coefficient are utilized to display the robustness of the suggested model. The simulation results demonstrate that the suggested approach provides a consistent means of classifying and categorizing various cerebral hemorrhages. The study abbreviates the phrases “true negatives”,“true positives”, “false negatives” and “false positives’’ to TN, TP, FN and FP correspondingly. To study the experimental outcomes, the following metrics are assessed.

### Confusion matrix

An essential part of our performance evaluation is the utilization of confusion matrix. The confusion matrix allows for a brief review of the classification model’s efficacy by displaying the number of true positive (TP), false positive (FP), true negative (TN), and false negative (FN) predictions.

### Accuracy

The metric of accuracy is employed to evaluate the general truthfulness of the classification outcome and is represented by

8 $$\:\:\:\:\:\:\:\:\:\:\:\:Accuracy=\frac{TruePositive+TrueNegative}{TruePositive+FalsePositive+TrueNegative+FalseNegative}$$

### Precision

This metric is the ratio of all positive predictions to successfully predicted positive classifications. It can be acquired numerically through

9 $$\:\:\:\:\:\:\:\:\:\:\:Precision=\frac{TruePositive+TrueNegative}{TruePositive+FalsePositive}$$

### Specificity

A metric named as Specificity or True Negative Rate (TNR) can be used to evaluate the ratio of accurately categorized negative cases to all negative cases.

10 $$\:\:\:\:\:\:\:\:\:\:\:\:Specificity=\frac{TrueNegative}{TrueNegative+FalsePositive}$$

### Sensitivity

The ratio of accurately categorized positive instances to all positive cases can be evaluated using a statistic called Sensitivity or True Positive Rate (TPR).

11 $$\:\:\:\:\:\:\:\:\:\:\:\:\:\:\:Sensitivity=\frac{TruePositive}{TruePositive+FalseNegative}$$

### F1 score

Using the F1 score, a mathematical formula determines the harmonic mean of Precision and recall, or Specificity.

12 $$\:\:\:\:\:F1\:Score=\frac{2 \times TruePositive}{2 \times TruePositive+FalsePositive+FalseNegative}$$

### Dice similarity coefficient

The primary evaluation metric used to gauge the degree of agreement between the actual data and the prediction is the Dice Similarity Coefficient (Dice), a commonly used metric for segmentation assessments in where |R| represents the actual volume of an image and |P| represents the predicted volume of an image.

13 $$\:DSC\left(R,P\right)=\frac{2\left|R\cap\:P\right|}{\left|R\right|+\left|P\right|}$$

### Intersection over Union

Intersection over union, or the Jaccard index, is another crucial segmentation metric that establishes the accuracy of a prediction about a feature.

14 $$\:\:\:\:\:\:\:\:\:\:\:\:\:IOU(R,P)=\frac{|R\cap\:P|}{|R\cup\:P|}$$

### AUC-ROC curve

Using the AUC-ROC measure, one may evaluate the model’s strength to identify the positive and negative examples. The true positive rate (recall) and false positive rate (specificity) at different categorization thresholds are compared in the ROC curve. The region under this curve, or AUC-ROC, provides an identifier that indicates the discriminative power of the model. Perfect classifiers ideally have an AUC-ROC value closer to 1, while AUC-ROC values of 0.5 are suggestive of a random or inefficient classifier.

## Experimental results

This section outlines our experimental design for the study which includes detailed evaluations of multiple evaluation measures, including recall, precision, accuracy, F1-score, dice, and IoU as well as a comprehensive assessment of our Spatial Attention-based CSR Unet model. We also demonstrate the visual representation of confusion matrices, loss curves, and receiver operating characteristic (ROC) curves.

### Environment for simulation

Considering the challenges and demands of intense computing we implemented the work in PyTorch and trained in a workstation with an elegant Graphics card NVIDIA GeForce RTX 3070 Ti paired with 32GB GDDR6X memory comprised of 192 tensor cores to handle the complex deep learning models. To improve the training results and reduce training durations, all images were resized to 256 × 256 pixels. During the training phase, the Adam optimizer helps to optimize the model parameters. We initially established a learning rate of 0.0001. After selecting the model with the greatest performance, a maximum of 60 training epochs is implemented. The hyperparameters and the training options are displayed in the Table 3.

The training procedure is predicated on the selection of model batches and initial weights, facilitating experiment replication and providing a precise representation of the enhancements achieved through hyperparameter adjustments. Before beginning the hyperparameter tuning procedure, it is imperative to clarify a few of the rows in Table 3.

The performance of learning algorithms is significantly influenced by hyperparameters, whose values change based on the particular problem area in which the algorithm is used. As a result, they need to be optimized for each dataset. It makes sense that the likelihood of obtaining a better-performing model increases with the number of hyperparameter combinations, but at the same time, they tend to greater computing costs. The number of epochs, the batch size, and other commonly used DL terms are included in these rows. The technique for optimizing the architecture weights was finally decided to be Adam Gradient Descent. Additionally, the Adam optimizer [61], renowned for its consistent performance on a range of tasks, was employed as part of the optimization approach to update the parameters of our model during training. Adam calculates and modifies learning rates by distinct features. It uses momentum in addition to storing a declining average of previous gradients to compute current gradients, in contrast to other approaches. The Adam algorithm has a good learning rate that does not disappear, low variance, and quick convergence. Furthermore, applying a dropout strategy proves to be efficient in enhancing the accuracy of attention-based processes.

To achieve the target classification performance level, we first heuristically determined the initial learning rate that allows for both the training and testing sets as indicators. We then used the same method to determine the learning rate drop factor, which allowed us to maximize the completion of the cost function minimum with elapsing epochs from the first training phase. We also determined after how many epochs the learning rate should decrease. Reducing it too soon after a few cycles might lead to virtually minimal network weight updates. On the other hand, waiting too long may cause the weights to continuously bounce toward the minimum of the cost function but never reach it.

### Training, testing of spatial attention-based CSR Unet model

To evaluate the model’s training processes, the model loss with accuracy validation methodologies are applied. The quantity of training iterations needed to extract features and transfer them to the subsequent learning layers is known as an epoch. During the training phase, if the model’s accuracy decreases and its loss increases, it indicates overfitting and is not learning. The indication shows that the model is learning when accuracy rises and loss falls.

The input images are 256 × 256 in size. Throughout the experiment, we used a batch size of 16 and a learning rate of 0.0001 with the Adam optimizer approach.

**Fig. 9:**
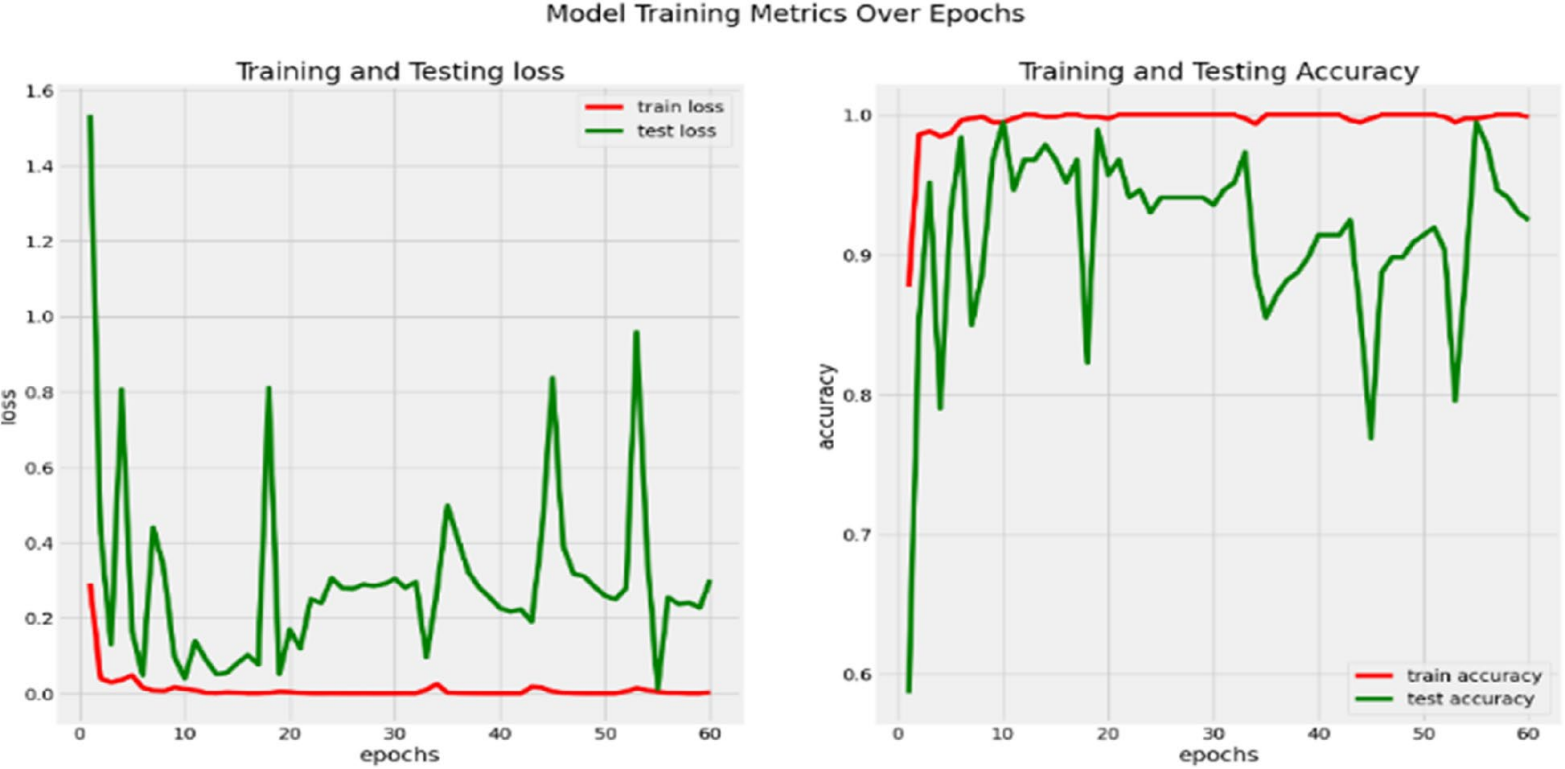
Model training, testing graph in loss & accuracy

The accuracy, loss of the proposed model are displayed for the train and test data in Fig. 9. For training and testing, a total of 828 images (training) and 46 images (testing) were used in the proposed model, which was given sixty epochs to complete. The model performed reasonably well in both training and testing, as seen by the experimental findings during segmentation. With sensitivity and specificity hitting 0.98 and 0.985, respectively, for an epoch count of 60, the training and testing accuracy were 99.83 and 98.34, respectively for the segmentation process.

### Resizing, CLAHE, and gamma results

**Fig. 10:**
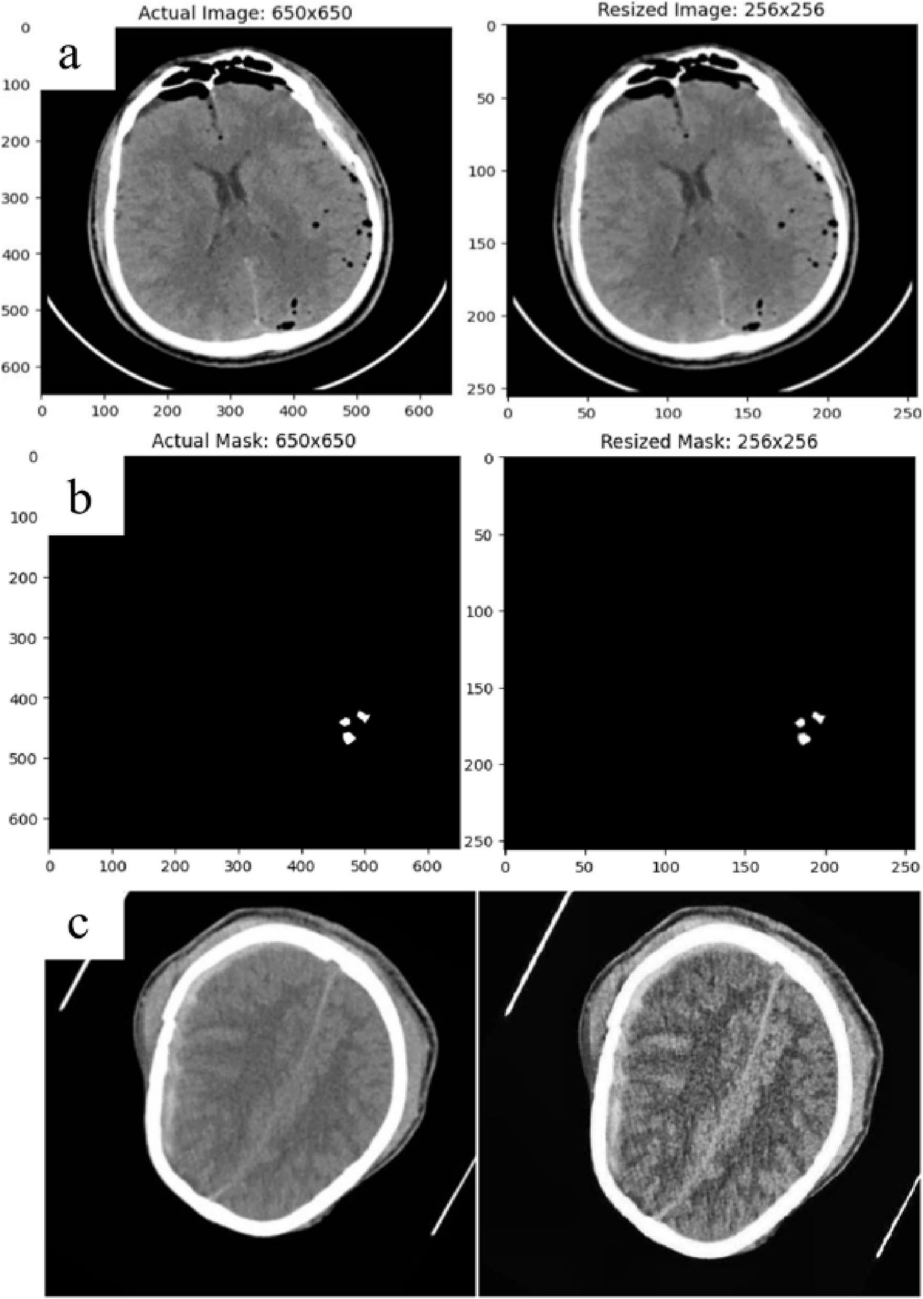
(**a**) Actual image comparison with a resized image (**b**) Actual mask comparison with a resized mask (**c**) Actual image comparison with CLAHE & Gamma applied image

The same-dimensional images must be used to train the deep learning models. By reducing images to the proper sizes, the learning process is expedited and the risk of overfitting is lower. Loss of data deteriorates the accuracy rate and model performance, making scaling images one of the more challenging tasks. A 256 × 256 image has been created by scaling down the original 650 × 650 image and mask sizes. The chosen dimensions for the proposed study were 256 × 256. It takes care of the issues of overfitting and rapid learning rate. The comparison of the original and resized image and mask is shown in Fig. 10.

### Segmentation results

**Fig. 11:**
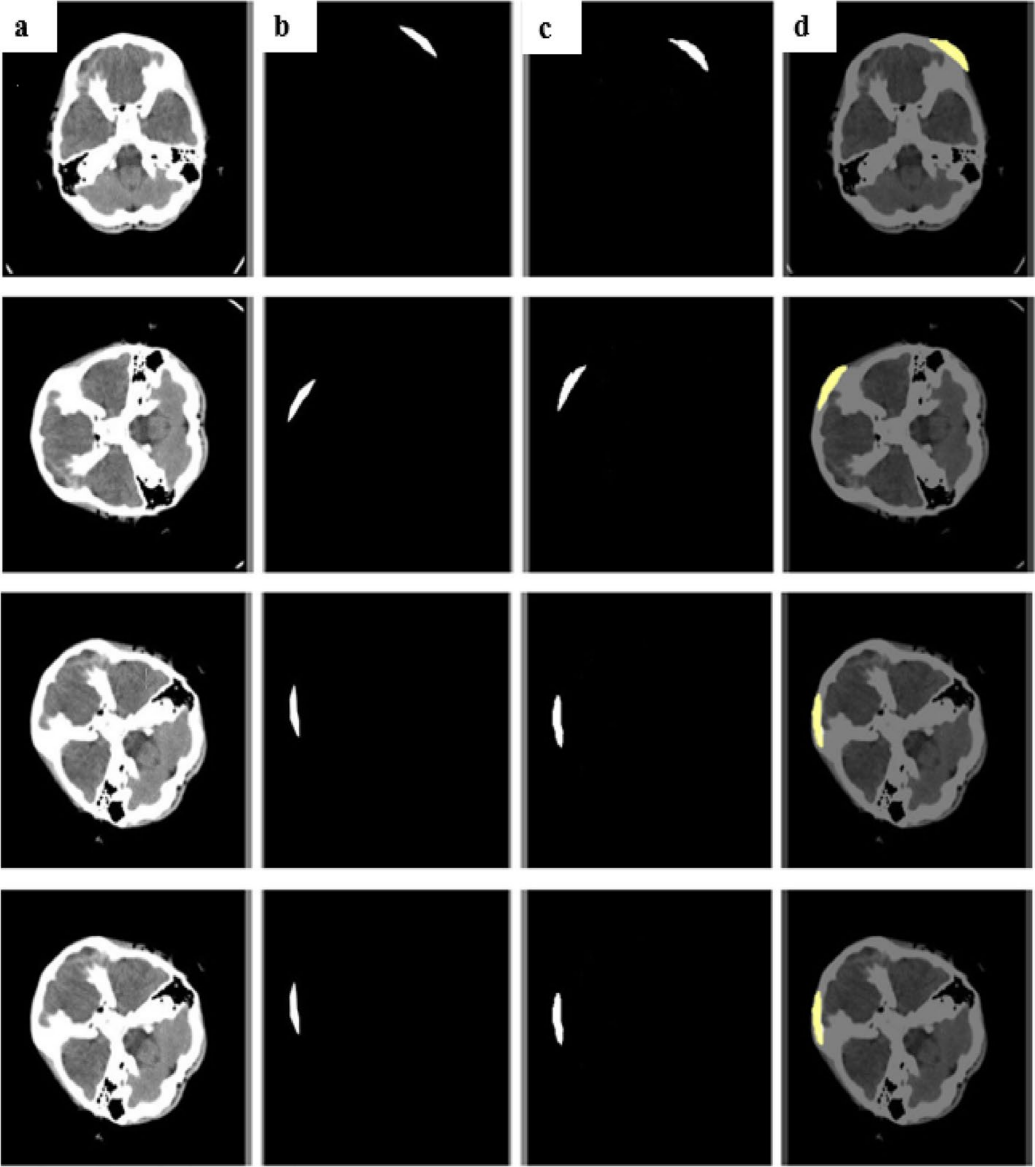
Depicts the segmented instances of EDH represented with input, ground truth, and prediction along with an overlaid image are given as (**a**)input image of EDH (**b**) ground truth (**c**) prediction (**d**) overlaid image

**Fig. 12:**
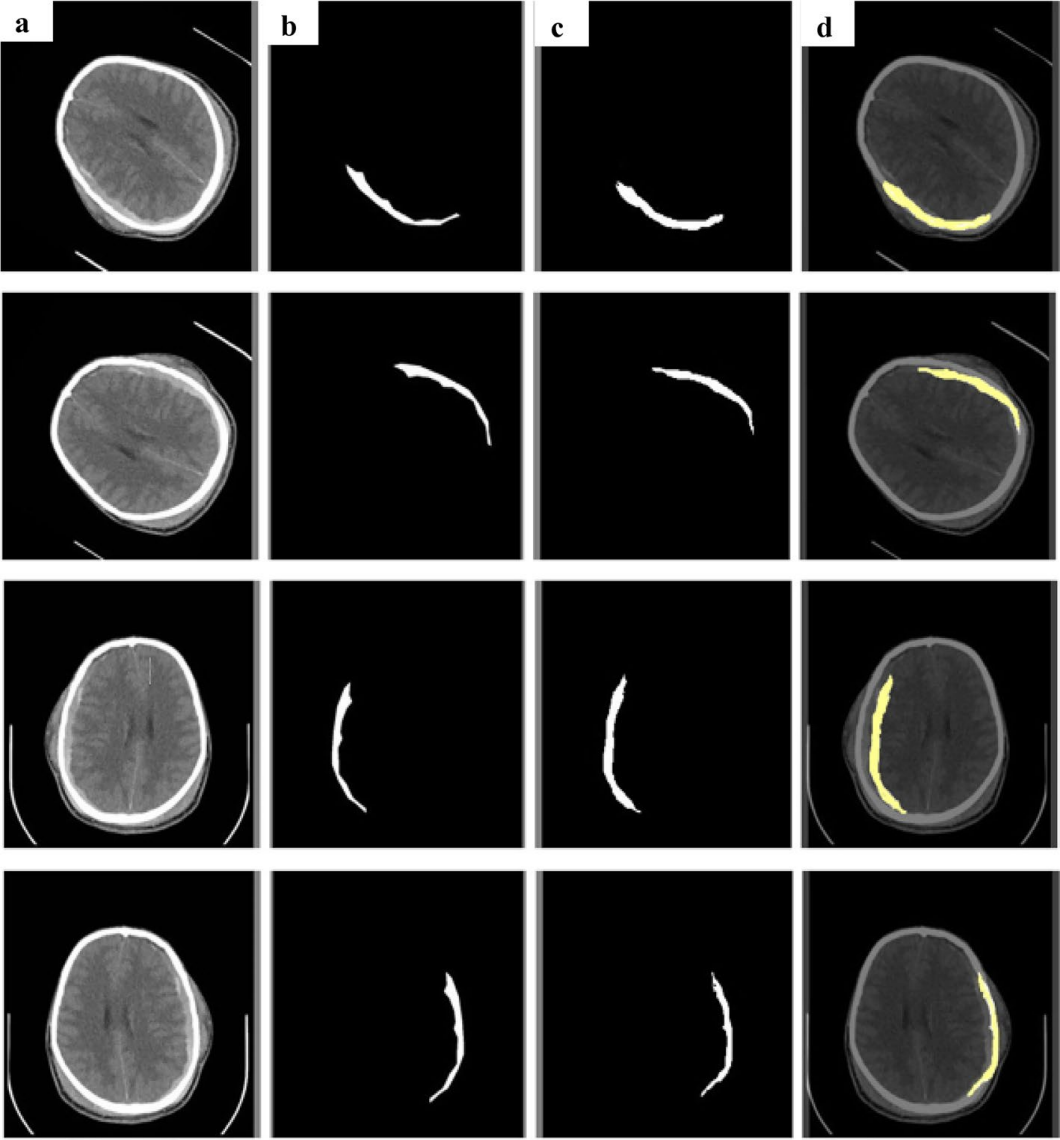
Depicts the segmented instances of SDH represented with input, ground truth, and prediction along with an overlaid image are given as (**a**) input image of SDH (**b**) ground truth (**c**) prediction (**d**) overlaid image

The segmentation approach used by Spatial Attention based CSR Unet was experimentally assessed for two classes using the physionet dataset. Figures 11 and 12 illustrates the comparison and assessment of the segmentation images, ground truth, predicted masks with their corresponding overlaid image for each class. We divided the dural hemorrhages in the experimental study and used the Dice coefficient and IOU to evaluate the model’s efficacy. The available dataset is used to evaluate the segmentation performance of the model and it has been trained with 828 images which were resized into 256 × 256 and preprocessed with enhancing techniques (CLAHE, SMOTE, and Gamma correction), though the 46 testing images were only resized and not enhanced by any technique. The Batch size was fixed to 32, the epoch count was set to 60, and learning rate was set to 0.0001.

According to the ICH sub-type results, the training dice scores of the proposed model, which provided the SDH and EDH segmentation, were 0.992 and 0.994, respectively (Table 4). The suggested segmentation strategy produced the testing dice values of 0.983 and 0.969 for EDH and SDH respectively, While the IoU for the training and testing data of SDH was 0.842 and 0.687, respectively, the IOU values for the training and testing data of EDH were 0.838 and 0.749, respectively.

**Table 4:** Dice coefficient and IOU values during ICH segmentation

Type of ICH	Dice coefficient (Training)	IoU (Training)	Dice coefficient (Testing)	IoU (Testing)
SDH	0.992	0.842	0.969	0.687
EDH	0.994	0.838	0.983	0.749

### Classification results

**Fig. 13:**
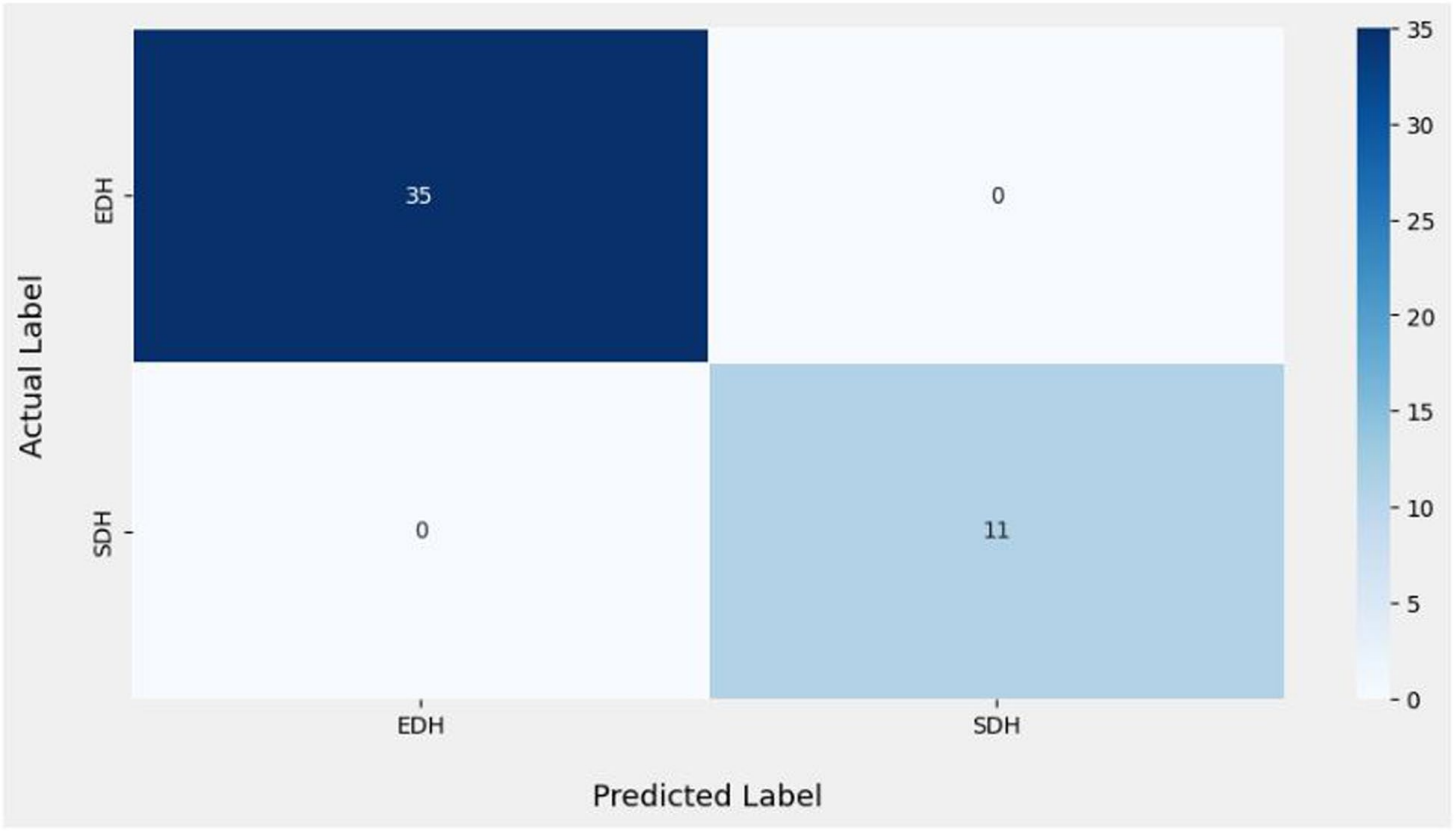
Confusion matrix for dural hemorrhage classification

The results of the dural hemorrhage classification based on their particular type are assessed in Fig. 13 which shows the confusion matrices of the suggested approach on 46 test images classified into two.

The confusion matrix presented in Fig. 13 notes two categories: the true class and the predicted class. It also gives metrics such as recall, precision, f1 score. The training and testing accuracy of the matrix were both 100%. Overall, the recommended model has a more satisfactory classification accuracy.

The techniques by which the SDH and EDH were correctly predicted have been identified, thanks to this categorization. The recommended method produces the fewest inaccurate estimations. The metrics for these two types of hemorrhage classification are shown in Table 5, along with the F1 score and precision. According to Table 5 below, the precision scores for both SDH and EDH are 1.0, respectively. Similarly, the recall values for both SDH and EDH are 1.0, even though the F1 score value reached the maximum value for both categories.

**Table 5:** Classification metrics of dural hemorrhages

Type of ICH	Accuracy	Recall	Precision	F1-Score
EDH	1.00	1.00	1.00	1.00
SDH	1.00	1.00	1.00	1.00
Macro avg	1.00	1.00	1.00	1.00
Weighted avg	1.00	1.00	1.00	1.00

The two types of hemorrhage classification provided extremely good and satisfactory classification accuracy, with 96% for SDH and 100% for EDH.

**Fig. 14:**
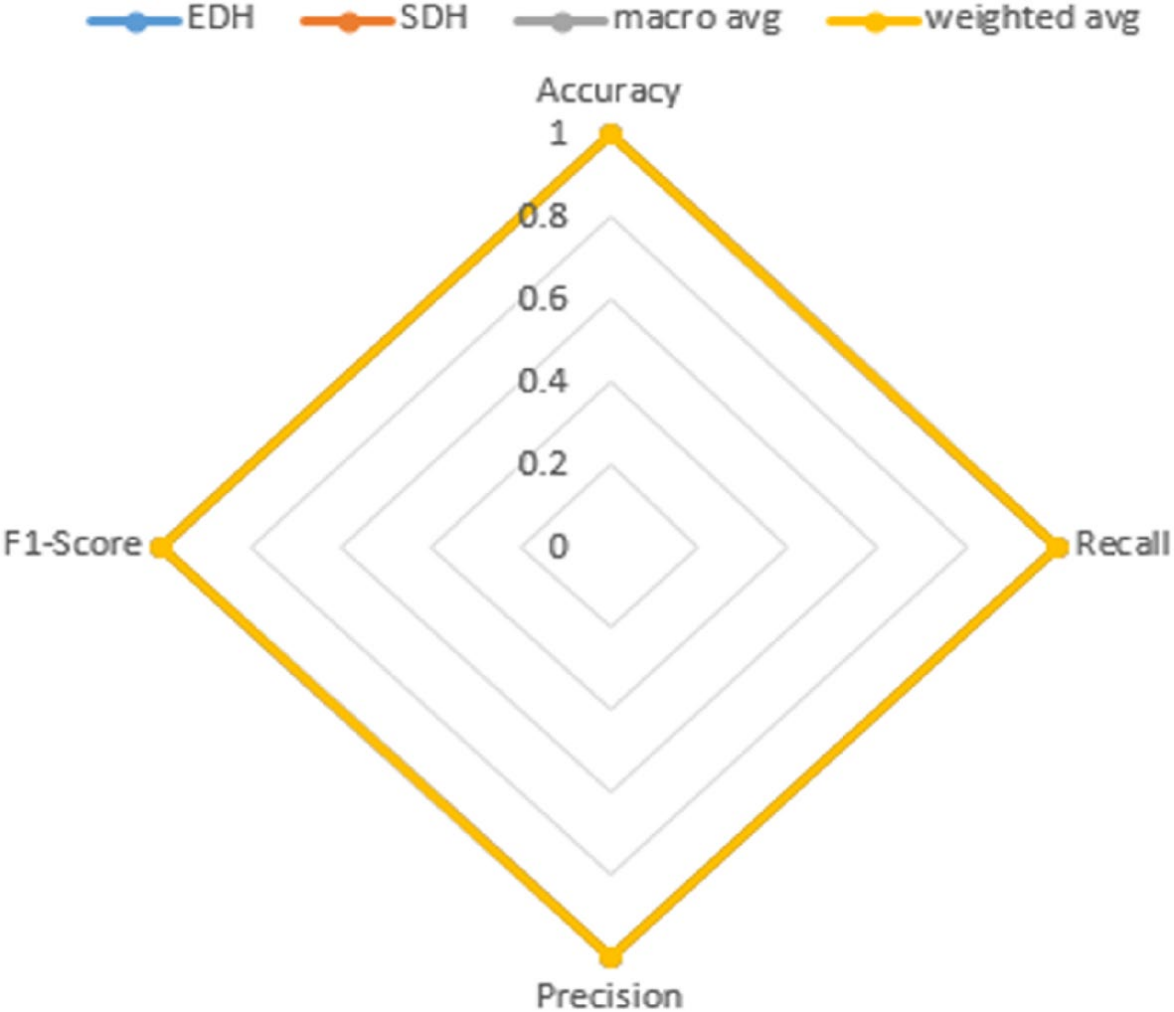
Precision, F1 -score, recall chart

**Fig. 15:**
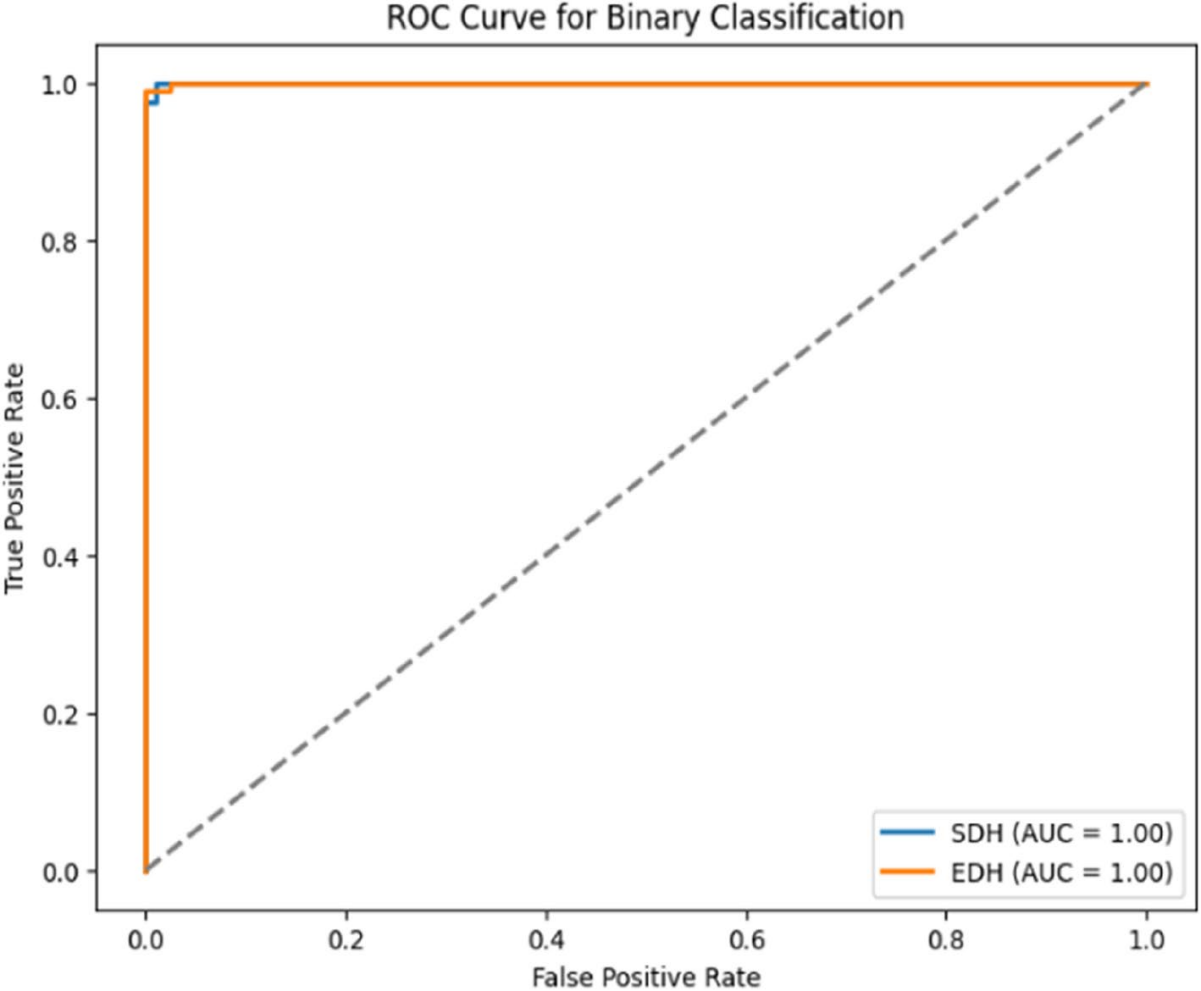
Binary ROC curve on the testing dataset

Figure 14 illustrates the chart view for the values obtained from the metrics Precision, Recall, and F1-Score. The metrics are represented by the blue line for Precision, the brown line for the F1-Score metric, and the green line for Recall. After categorizing all of the assessment indications of dural hemorrhages, we generated ROC curves with the ranges of uncertainty included, as illustrated in Fig. 14. where the ROC curve represents the baseline. By considering each of these curves, we were able to observe the variance in the curve while calculating the average Area Under the Curve (AUC) for each class, as shown in Fig. 15.

The Fig. 15 depicts the ROC curves produced for the subdural and epidural categories with both them reaching AUC value of 1.

## Discussion

The final goal of this research is to verify that the suggested model outperforms the most advanced deep learning techniques in terms of segmenting ICH and categorizing the hemorrhages of dural subtypes. The Spatial Attention based CSR Unet model’s specificity, sensitivity, and accuracy in identifying the hemorrhage is improved. Compared to existing methods, the proposed model performs significantly better in segmentation, specifically targeting the two dural subtypes, and outperforms in terms of Dice coefficient, recall, precision, F1 score. Considering the segmentation visualization, the proposed model’s segmentation results are more consistent with detailed and precise feature extraction. As a result, there is a reduced likelihood that the textures will be wrongly identified as either type of the dural hemorrhages SDH and EDH.

Moreover, our model shows higher sensitivity in locating the bleeding site compared to other deep learning methods. Consequently, since the proposed Spatial Attention based CSR Unet model can more successfully separate different ICH regions from CT images, there might be potential for practical application. Our dataset contained only 229 dural slices because 36 of the 82 patients were diagnosed with ICH. We employed the SMOTE technique to avoid class imbalance issues in the data. Tables 6 and 7 display the performance comparison of the suggested and current approaches/models during segmentation and classification. The Table 6 deals with the comparison of the classification metrics of the proposed model Spatial attention-based CSR Unet with other current models/methods. The overall accuracy of the proposed model was very good with a score of 100 which outperforms other models like Modified CNN + VGG16 and attention-based CNN. Even the sensitivity and specificity values of 100 for both categories were very much satisfactory compared to the other models.

**Table 6:** Comparison of the current approaches with proposed model metrics during classification

Authors	Model/Method	Dataset	Training: Testing ratio	Accuracy %	Specificity	Sensitivity	Precision	Recall	F1-Score
				Overall					
Nizarudeen et al., 2023 [62]	DenseNet-ResNet + IRF	Physionet	75:25	98.5	98.50	-	97.90	99.05	97.50
Phaphuangwittayakul et al.,2022 [38]	EfficientNet-B0-B4	Physionet	80:20	98.41	99.71	72.09	92.54	-	81.05
Hilal et al., 2022 [63]	Efficientnet + FLNN	Physionet	80:20	97.77	98.48	94.37	94.73	-	-
Venugopal et al., 2021 [64]	FFEDL-ICH	Physionet	80:20	96.56	97.93	95.65	96.43	-	-
**Proposed**	**Spatial attention-based CSR Unet**	**Physionet**	**80:20**	**100**	**100**	**100**	**100**	**100**	**100**

**Table 7:** Comparison of the current approaches with proposed model metrics during segmentation

Authors	Model/Methods	Training: Testing ratio	Dataset	Dice Coefficient	IoU
Xiao et al., 2024 [65]	MPFR-Net	80:20	Physionet	0.704	0.704
Ma et al., 2023 [66]	IHA-Net	80:20	Physionet	0.869	0.605
Wang H et al., 2023 [67]	MSRL-Net	80:20	Physionet	0.717	0.596
Mehendale et al., 2022 [68]	CNN + GNN	80:20	Physionet	0.813	-
**Proposed**	**Spatial attention-based CSR Unet**	**80:20**	**Physionet**	**0.970**	**0.718**

Additionally the Table 7 comprises of the segmentation metrics of the proposed model Spatial attention based CSR Unet with other current models/methods. The mean dice coefficient was outperformed by the proposed model with a score of 0.970 and even the IoU values are satisfactory with SDH and EDH reaching 0.68 and 0.74. The low IoU value of SDH compared with EDH might be the larger hemorrhagic regions in SDH where the segmented parts might have suffered lower intersection, even though the proposed model outperforms the other suggested models in comparison.

The improved results for dural segmentation and classification that the proposed model has produced are shown in Tables 6 and 7. By using the Spatial Attention-based CSR Unet approach, which makes the convolutional layers recognize and concurrently calculate better features across several levels, detailed features to coarse features have been incorporated. The process involves computation of the fine features specified by the layers of the proposed model. A network branch with completely linked layers is then applied for classification comprising detailed feature maps that are concatenated after upsampling.

With much more satisfying accuracies of 1 for both EDH and SDH, the proposed methodology successfully classified diseases with a classification accuracy of 1 for the testing set, and during the segmentation process the mean dice coefficient of 0.970 and 0.718 IoU resulting in the testing set. The tissues covering the ventricles of brain could be an issue, as they could have impacted the epidural type’s accuracy. This issue was unfolded when we carried out in a previous work (under review) on the segmentation and classification of five types of hemorrhages namely IVH, IPH, SAH, SDH, and EDH, which motivated us to concentrate on only two types of dural hemorrhages. The collected data show that compared to the other models and methods in the table, the recommended model outperformed them. Even though in the case of training set, the segmentation dice coefficient for EDH is 0.994, but the value for SDH is 0.992, since the subdural type is close to the skull, it can be challenging to distinguish lesions when they first appear. Additionally, lesions often have an uneven shape and spread into the sulci, hence this factor contributes to the SDH’s slightly worse performance than the other category epidural. Nonetheless, the Spatial Attention-based CSR Unet can more accurately describe the interface between the two types of bleeding at that location.

When dural types (SDH, EDH) are present, the Spatial Attention-based CSR Unet reduces the likelihood of misinterpreting a skull for bleeding and texture issue causing misidentification of EDH as SDH and vice versa. Additionally, with a solid and satisfactory IoU score of 0.718 for the testing set, our approach is able to segment and detect dural hemorrhages more sensitively, while classification has provided exceptional results with an accuracy of 100%. However, the test data is not meant to be utilized for training, rather its sole function is to measure its quality, meaning that the network is not anticipated to gain any knowledge from it. It should only be used to modify parameters as an alternative by considering the testing measurements, which can be less than the training metrics of many models. Furthermore, there are several limitations in this work where the dataset available in Physionet has limited images and masks where deep learning algorithms are data hungry, this limited dataset can provide limited features to learn from the images and masks available, Even though there are plenty of segmentation datasets which are publicly available but they lack the masks of the corresponding images hence we couldn’t compare this work with other types of brain hemorrhage datasets. A diversified dataset would have been much better for comparing and verification. Computational complexity and memory requirements can consume more time, particularly when working with enormous images or with constrained hardware where enhanced optimization may require vast tuning of hyperparameters. Even though the fact that the attention mechanism and residual connections have been proven to be efficient for preserving feature diversity and improving overall performance on smaller datasets which is aligned with our objective, it is still uncertain about its suitability towards larger datasets and more variety of models.

## Conclusion

The death rate from ICH, a dangerous medical disease is extremely high. To develop treatment and surgical tactics, CT scans of patients who may have dural hemorrhages must be segmented and identified. Despite the severity of this concern, there are only a few trustworthy procedures for ICH segmentation that don’t include a medical specialist. Therefore, accurate and dependable methods for removing dural regions from CT scans is crucial and needs more development. Regarding the separation of intracranial hemorrhages from CT scans, deep learning algorithms present challenges due to their poor resolutions and considerable heterogeneity in hemorrhagic location, contrast, and morphology. Our study on deep learning algorithms for ICH segmentation has provided us with important insights. We proposed a deep learning strategy called Spatial Attention-based CSR Unet framework for segmentation and classification. The model’s capacity to learn a variety of feature representations as the network gets deeper for downstream prediction is improved by residual connections, which provide a direct channel for features from earlier layers to guide deeper layers. With Squeeze Excitation involved with the residual connection it helps in learning deep features and guides towards rich feature extraction. In order to aid in the prediction and extraction of the precise regions of features, Spatial Attention- based CSR Unet is implemented with the help of squeeze excitation and spatial attention modules. This multiple-branching architecture satisfies the need for more accurate segmentation with improved feature mapping and may also be utilized to identify the various categories inside the region of interest. To predict the microscopic bleeding patches, image enhancement is crucial, and gamma correction and the CLAHE approach aid in this process. A key component in classification of dural hemorrhages are the fully connected layers of the proposed model. There would be fewer false-negative hemorrhage detections if these algorithms could outperform subject-matter experts once their accuracy rates are sufficiently high. Treatment delay puts a patient with a dural form of bleeding at risk of death. It matters a great deal how long it takes between symptoms and diagnosis. Therefore, utilizing these contemporary methods can help shorten this time. The developed biomedical image-based hemorrhage detection model can be very helpful to doctors and other healthcare professionals when applied in hospitals and medical settings.

## Data Availability

Data will be shared by the reasonable request.

## Abbreviations

ICH: Intracranial Hemorrhage
CT: Computed Tomography
DICOM: Digital Imaging and Communications in Medicine
SDH: Subdural Hemorrhage
EDH: Epidural Hemorrhage
CSR: Convolution Squeeze Excitation and Residual
SAM: Spatial Attention Module
AI: Artificial intelligence
AUC: Area under the curve
FC: Fully connected
ROC: Receiver operating characteristic
GPU: Graphical processing unit
CLAHE: Contrast Limited Adaptive Histogram Equalization
SMOTE: Synthetic minority oversampling technique
ReLU: Rectified Linear Unit
BN: Batch normalization
DSC: Dice Similarity Coefficient
IOU: Intersection over Union

## Footnotes

1 United Nations DoEaSA, Population Division (2019) World Population Prospects 2019: Methodology of the United Nations population estimates and projections (ST/ESA/SER. A/425).

2 *Computed Tomography Images for Intracranial Hemorrhage Detection and Segmentation v1.3.1 (physionet.org)*.
